# Reactive Extrusion Synthesis of Biobased Isocyanate-Free Hydrophobically Modified Ethoxylated Urethanes with Pendant Hydrophobic Groups

**DOI:** 10.1021/acssuschemeng.2c03535

**Published:** 2022-08-22

**Authors:** Dominik Wołosz, Aleksandra Marta Fage, Paweł Grzegorz Parzuchowski, Aleksandra Świderska, Robert Brüll

**Affiliations:** † Warsaw University of Technology, Faculty of Chemistry, Noakowskiego 3, 00-664 Warsaw, Poland; ‡ Fraunhofer Institute for Chemical Technology ICT, Joseph-von-Fraunhofer-Straße 7, 76327 Pfinztal, Germany; § Fraunhofer Institute for Structural Durability and System Reliability LBF, Bartningstraße 47, 64289 Darmstadt, Germany

**Keywords:** rheological thickeners, hydrophobically modified ethoxylated urethanes, isocyanate-free polyurethanes, fatty acid derivatives, reactive extrusion, bis(cyclic carbonate)

## Abstract

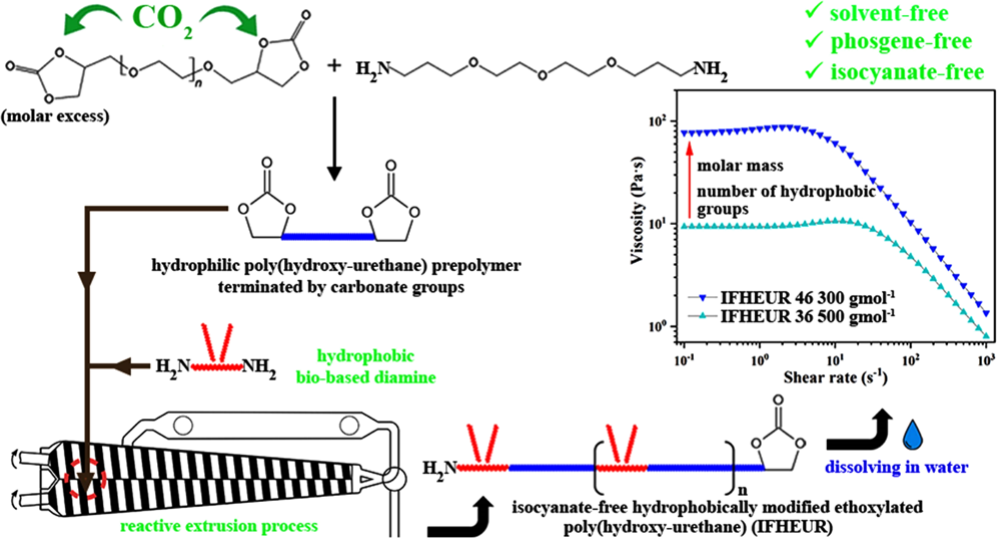

Development of hydrophobically modified ethoxylated urethane (HEUR) rheology modifiers enabled the widespread application of waterborne paints and coatings, replacing their environmentally burdening solvent-based predecessors. However, the diisocyanates, required for the conventional synthesis of HEURs, pose severe eco-sustainability threats. In this paper, we demonstrate an innovative approach to avoiding toxic components in the preparation of rheology modifiers by obtaining a new class of water-soluble isocyanate-free hydrophobically modified ethoxylated poly(hydroxy-urethane)s (IFHEURs). The first step in the synthetic pathway was the preparation of CO_2_-based five-membered poly(ethylene glycol) bis(cyclic carbonate) and its subsequent aminolysis using 4,7,10-trioxa-1,13-tridecanediamine, yielding poly(hydroxy-urethane) (PHU) prepolymers terminated with cyclic carbonate groups. The PHU prepolymers were further extended in a reactive extrusion (REX) synthesis using biobased hydrophobic diamine PRIAMINE 1075. The REX technique made it possible to overcome the typical limitations of the aminolysis reaction and to reach the desired conversion within a moderate reaction time. IFHEURs have been structurally elucidated using FT-IR and NMR spectroscopy techniques, MALDI-ToF mass spectrometry, and SEC analysis and applied as rheology modifiers. The study of their associative behavior in aqueous solutions confirmed that the architectural flexibility of the obtained IFHEURs, containing terminal and pendant hydrophobic groups, opens a perspective for tuneable thickening performance.

## Abstract 2

Biobased hydrophobically modified ethoxylated poly(hydroxy-urethane)s have been synthesized without the toxic isocyanate components using the efficient reactive extrusion method.

## Introduction

Waterborne paints^1^ and coatings^2^ are steadily replacing solvent-based formulations due to environmental and health concerns and the consequent requirement to reduce the use of volatile organic compounds in commercial products. The transition toward more sustainable, water-based alternatives was achieved without compromising the desired performance of these products by incorporating rheology modifiers. The essential role of these modifiers is to drive the viscosity profile and, thus, to adjust the behavior of the paint during both storage and application. The market for rheology modifiers has grown constantly in the last decade and is expected to reach $6.83 billion by 2024.^3^

Hydrophobically modified ethoxylated polyurethanes (HEURs) are a representative class of polyurethane (PU) rheological thickeners. They are water-soluble associative polymers consisting of a hydrophilic backbone and hydrophobic terminal groups.^3−5^ Typical HEURs are oligomeric molecules with a hydrophilic core based on poly(ethylene glycol) (PEG) with a molar mass in a range of 6,000–35,000 g·mol^–1^ and short hydrophobic terminal chains.^3,6^ Hydrophobes most often contain C8–C18 alkyl groups,^2,7,8^ alkyl phenyl groups,^9−12^ or fluorocarbons.^13,14^ The hydrophilic segment of HEURs is typically obtained via polyaddition of PEG^2,3,7^ and diisocyanate (e.g., isophorone diisocyanate^2,4,10,12,15−18^ or 4,4′-methylenedicyclohexyl diisocyanate^3,8,19^), used in molar excess and yielding a telechelic prepolymer, terminated by isocyanate groups. In a subsequent step, the prepolymer is end-capped with hydrophobic alcohol or amine to obtain the amphiphilic HEURs.

Due to their telechelic architecture, HEURs form a transient network in an aqueous solution, which exhibits a complex rheological response under stress. The self-assembly mechanism of HEURs is based on a micellar aggregation of the hydrophobic tails.^3,6,20^ The polymer chains link micelles together and form a three-dimensional network, manifested by a macroscopic increase of the solution viscosity.^21^ These intermolecular junctions can be reversibly disrupted, reducing the viscosity of the system under shear. In this state, the hydrophobic tails agglomerate in a core of isolated micelles, while the hydrophilic chains are coiled, creating flowerlike loops. Thus, the thickening effect of the physically cross-linked HEURs in waterborne paints determines their shelf-life and prevents sedimentation of additives. Furthermore, the fluent and reversible shear thinning enables easy application of paint, limits the spattering behavior, and simplifies its levelling.^3−5^

For linear, end-functionalized HEURs, the relationship between the structure and the mechanism of self-assembly in aqueous solutions is well understood. The architectural factors such as the kind of hydrophilic core^20,22^ and hydrophobic terminal groups^7,12,23,24^ and molar mass^4,8,25−27^ of the HEURs have an interdependent influence on their rheological properties. The density of the network and the resulting thickening effect of HEURs at a given concentration in an aqueous solution are determined by the ratio of the length between the hydrophilic segment and the hydrophobic end chains.^3^ An optimal ratio between these building blocks leads to a predominating intermolecular association mechanism and a dense network. At a constant length of the hydrophobic end-capper, elongation of the hydrophilic backbone above that optimal threshold weakens the network. Furthermore, the density of the micellar junctions in the solution decreases if the length of the hydrophilic segment is inadequate to connect the adjacent micelles to a high extent. Instead, the intramolecular associations are dominant. However, if the concentration of such short HEUR molecules is sufficient for the chains to reach the neighboring micellar junctions, they form a highly dense network.^3^

Although studies on the structure–property relationship mostly address telechelic HEURs, end-capped with single-tail hydrophobes, few analogous structures containing multiple-tail hydrophobes or branched architectures have been reported.^10,11^ The exemplary case are HEURs based on different Percec-type alkyl-substituted benzyl alcohol dendrons as hydrophobic end-cappers, bearing one, two, or three hydrophobic alkyl chains.^11^ These dendron HEURs showed gradually developing associative networks in an aqueous environment as a function of increasing numbers of hydrophobic tails. The HEURs containing 4-mono(decyloxy)benzyl alcohol formed mainly isolated micelles and behaved as Newtonian fluids in a wide range of shear rates, whereas HEURs obtained from 3,4,5-tri(decyloxy)benzyl alcohol built a strong physical network and exhibited clear shear thinning behavior throughput the whole range of the studied shear rate. A higher number of hydrophobic dendron tails changed the rheological behavior of the solutions from viscous fluids to viscoelastic fluids and finally to an elastic body. In principle, the incorporation of hydrophobes through pendant chains, multiarm structures, or comblike structures opens further possibilities to tune the performance of HEURs. However, it also poses challenges related to controlling the architecture. Random or insufficient positioning of the hydrophobic centers along the hydrophilic backbone causes the formation of inhomogeneous networks in an aqueous solution, which are less resistant to fracture.^28^ For this reason, the perspective of reaching new properties of HEUR through innovative structures requires reliable synthesis protocols, allowing the design of the distribution of the associative groups.

**Figure 1:**
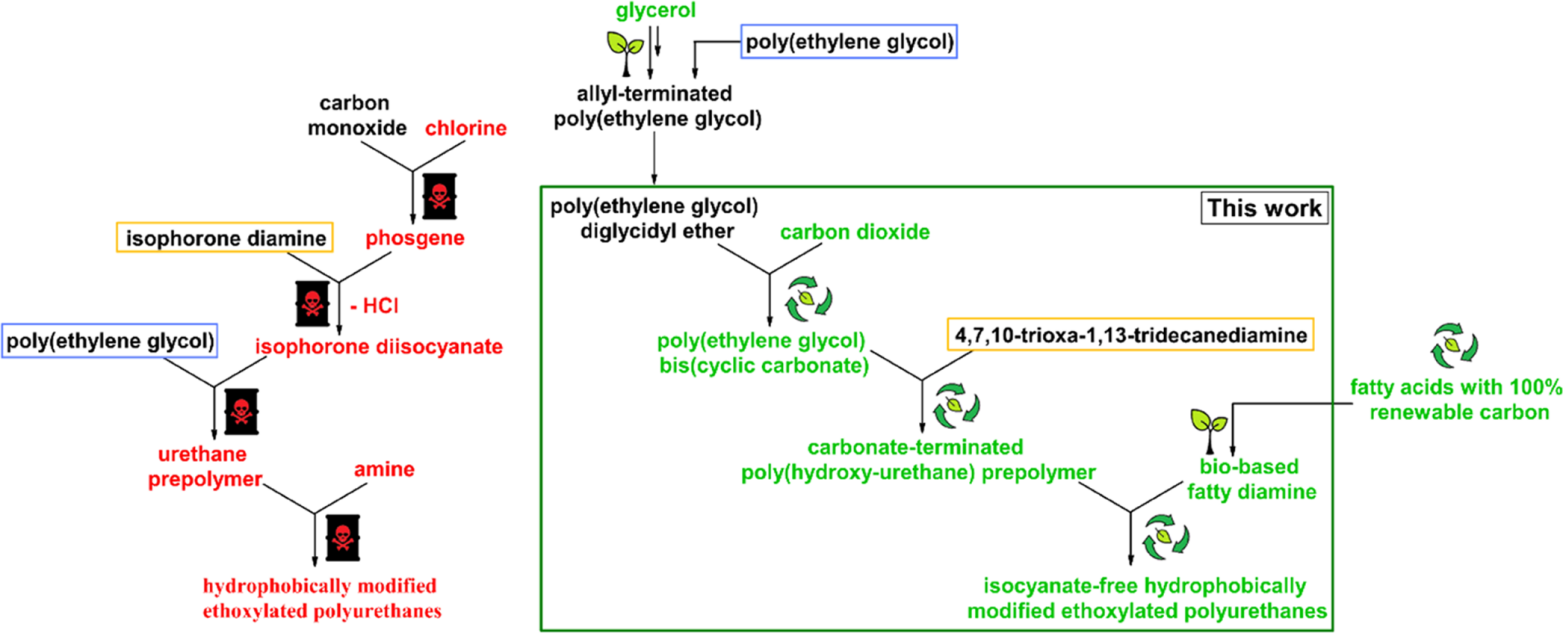
Synthesis trees for the production of standard HEUR associative rheology modifiers (left) and sustainable isocyanate-free HEUR alternatives (right).

The extensive research efforts aim to improve the efficiency of rheological thickeners; however, the impact of HEURs themselves remains unaddressed, despite significant environmental issues concerning the application of toxic isocyanates during their synthesis. An exemplary manufacturing pathway of a standard HEUR product, presented in Figure 1 (left), illustrates several critical steps. Aside from the harmful properties of the diisocyanate components, their synthesis is carried out using a hazardous process of phosgenation of aliphatic or aromatic primary diamines,^29^ which first requires the use of chlorine for the production of phosgene. The phosgenation step releases significant quantities of corrosive hydrochloric acid, which requires large amounts of wastewater for its disposal.

A promising alternative to replace the isocyanates in the synthesis of HEURs is direct use of amines through polyaddition with five-membered cyclic carbonates, forming poly(hydroxy-urethane)s (PHUs).^30^ An appropriate selection of cyclic carbonates and diamines with an oxyethylene core analogous to PEG would enable the formation of a water-soluble segment, similar to the conventional, isocyanate-based HEURs. The structure of such isocyanate-free, hydrophobically modified ethoxylated poly(hydroxy-urethane)s (IFHEURs) would thus differ from standard HEUR only in the presence of free hydroxyl groups formed at each urethane bond, further increasing the hydrophilic nature, while the applicability of the hydrophobic amine end-cappers remains unchanged. It can be thus anticipated that in terms of recyclability, e.g., by solvolysis, IFHEURs would be comparable to the conventional PU-based additives. However, a straightforward development of PHU-based alternatives is limited due to the relative novelty of the approach, restricting access to commercially available cyclic carbonates. Furthermore, only a few studies focus on increasing the efficiency of solvent-free aminolysis of cyclic carbonates, which typically suffers from long reaction times (hours to days), insufficient conversion of cyclic carbonate groups, and weak homogeneity of the product. It is therefore challenging to obtain high-molar-mass PHUs using a bulk polymerization process in a typical batch reactor.^30−34^ It was, however, demonstrated that the kinetic limitations of the PHU reaction can be overcome in a solvent-free approach using the reactive extrusion (REX) technique.^31^ Schmidt et al. presented the PHUs obtained via the REX process for the first time in 2017.^35^ The particular advantages of the REX process were shown by Magliozzi et al., who studied the synthesis of diglycerol bis(cyclic carbonate)-based PHUs in a laboratory extruder.^31^ The REX eliminated the issues concerning the mixing of highly viscous melts, which are exacerbated in a PHU matrix by extensive hydrogen bonding and therefore greatly decreased the synthesis time compared to the bulk polymerization process in a batch reactor.^31^ Despite the capability of REX to tackle the challenges of melt polymerization, often exploited in industrial settings, research on using an extruder as a reactor for PHU synthesis is still rare.

**Scheme 1:**
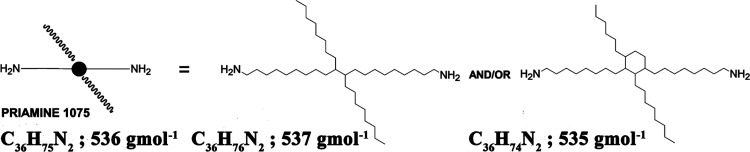
Schematical structure of PRIAMINE 1075 with pendant aliphatic chains corresponding to its possible molecular architecture

In this paper, we present a perspective for a new class of sustainable rheological thickeners and demonstrate a green REX method for their efficient synthesis without the use of toxic components. In contrast to conventional HEURs, halogen-containing compounds, phosgene, and diisocyanate were not used during the manufacturing process of IFHEURs, as illustrated in Figure 1 (right). The hydrophilic segments were obtained through the aminolysis of five-membered PEG bis(cyclic carbonate) (BCC) reacted with 4,7,10-trioxa-1,13-tridecanediamine (TTDDA) and forming cyclic carbonate-terminated PHU prepolymers with varying chain lengths. Thus, the PEG-based backbone is used in both conventional and newly proposed synthesis pathways, providing a hydrophilic nature, while in the case of PHU prepolymers, the diisocyanate component was replaced by a diamine. The green approach to the synthesis of IFHEURs was enhanced by the incorporation of a CO_2_ molecule to produce each cyclic carbonate group of BCC, which is subsequently built into the urethane bonds. For future developments, the functionalization of PEG with glycidyl groups, needed for the synthesis of BCC, can also be achieved starting from biobased glycerol, subsequently converted to allyl alcohol, allyl-terminated PEG, and finally to the PEG diglycidyl ether.^36−38^ Moreover, we carried out the hydrophobic modification toward IFHEURs by solvent-free REX using fatty diamine based on fully renewable carbon sources—PRIAMINE 1075, which contains two pendant aliphatic tails, as shown in Scheme 1. The chain extension of PHU prepolymers in varied formulations with the diamine formed strongly hydrophobic centers, distributed between the hydrophilic segments of the IFHEURs. Comprehensive spectroscopic and chromatographic analyses provided insight into the structural composition of the PHU prepolymers and the resulting IFHEUR products. The rheological study showed the correlation between the achieved molecular architecture and associative behavior in an aqueous solution, indicating the potential of the IFHEUR species for a new and improved approach toward rheological modifiers.

## Experimental Section

### Materials

Poly(ethylene glycol) (PEG) diglycidyl ether (approx. *M̅*_n_ = 500 g·mol^–1^, data provided by the supplier), 4,7,10-trioxa-1,13-tridecanediamine (TTDDA, purity 97%), potassium iodide, and 18-crown-6 were purchased from Sigma-Aldrich. Food-grade CO_2_ was purchased from Multax sc. PRIAMINE 1075 (PRI) (approx. *M̅*_n_ = 550 g·mol^–1^, data provided by the supplier) was kindly provided by Croda International Plc. All materials were used as received.

### Instrumentation and Measurements

FT-IR spectra were recorded on a Nicolet iS5 Mid Infrared FT-IR Spectrometer equipped with an iD7 ATR Optical Base.

^1^H NMR and ^13^C NMR spectra were recorded on a Varian VXR 400 MHz spectrometer using CDCl_3_ or DMSO-*d*_6_ as a solvent and analyzed with MestReNova. The integrals of signals in ^1^H NMR spectra of selected products were used to calculate their number-average molar mass (*M̅*_n(NMR)_) and the content of functional groups (eqs S1 and S3–S7).

MALDI-ToF mass spectrometry measurements were performed on a Bruker UltraFlex MALDI-ToF/ToF Spectrometer (Bremen, Germany) in a linear or reflection mode using 2,5-dihydroxybenzoic acid as a matrix and Bruker Peptide Calibration Standard (1047.19-3149.57 Da). The spectra were analyzed using Polymerix v.2.0 (Sierra Analytics Inc.) software.

Size-exclusion chromatography (SEC) measurements were performed using an SEC 1200 system with UV-G1314A at 254 nm and RI detector G1362A (Agilent Technologies, Agilent 1100 Series HPLC system). The column set consisted of five columns: PSS PFG 7 μm with 1000 Å, 300 Å and 2 × 100 Å (0.78 × 30 cm) and a PSS PFG 7 μm guard column (0.78 × 5.0 cm). The used eluent was DMAc with 50 mmol LiCl at 50 °C. The flow rate was 1 mL·min^–1^. Calibration was performed with PEG standards from PSS in the molar mass range of 12,600–108,000 g·mol^–1^. The concentration of the sample and the injected sample volume were 3 g·L^–1^ and 100 μL, respectively. The obtained chromatograms provided data on the number-average molar mass (*M̅*_n_), weight-average molar mass (*M̅*_w_), and dispersity (*Đ*_M_) of selected products.

The rheological behavior of the obtained polymers in 10 and/or 20 wt % aqueous solutions was characterized on an MCR 501 rheometer (Anton Paar Germany GmbH) with a parallel plate-plate geometry (diameter 25 or 40 mm, gap 0.5 mm) at 25 °C. Steady shear and oscillatory measurements were carried out to determine the viscosity profiles in dependence on the shear rate and the viscoelastic properties, respectively.

### Synthesis of Poly(ethylene glycol) bis(cyclic carbonate) (BCC)

The synthesis of BCC was carried out according to a modified literature procedure.^39^ The PEG diglycidyl ether (290 g, 0.58 mol) was introduced into a stainless-steel autoclave (500 mL) equipped with a temperature gauge, manometer, gas inlet port, magnetic stirrer, and a heating bath (Wood’s alloy). Subsequently, the catalysts potassium iodide (KI) (0.15 g, 0.0009 mol) and the 18-crown-6 (0.08 g, 0.0003 mol) were added to the autoclave, which was then sealed and purged with CO_2_ to remove the air. Next, the CO_2_ (59 g, 1.34 mol) was introduced into the reactor and the synthesis was carried at 140 °C for 72 h, under continuous stirring at 700 rpm. Finally, the autoclave was cooled to room temperature, the unreacted CO_2_ was removed, and the autoclave was opened. The BCC product was obtained as a clear liquid in a high yield (324 g, 0.57 mol, yield = 98%). Its structural analyses are presented in the Supporting Information (Figures S1–S3). The *M̅*_n(NMR)_ of BCC was estimated at 570 g·mol^–1^ and used for further stoichiometric calculations in the synthesis of prepolymers (Figure S2 and eq S1).

^1^H NMR (400 MHz, CDCl_3_): δ (ppm) = 3.62–3.74 (m, CH_2_CH_2_O and CH_2_O, 35.89 H), 4.39 (m, CH_2′_O, 2 H), 4.49 (t, *J* = 8.0 Hz, CH_2″_O, 2 H), 4.82 (m, CH–O, 2 H). ^13^C NMR (100 MHz, DMSO-*d*^6^): δ (ppm) = 66.07 (CH_2_–O), 69.85 (CH_2_–O_(cyclic)_), 75.61 (CH-O_(cyclic)_), 155.01 (C=O). FT-IR (ATR): 2870, 1791, 1463, 1352, 1083 cm^–1^.

### Synthesis of Hydrophilic Cyclic Carbonate-Terminated Poly(hydroxy-urethane) (PHU) Prepolymers

The syntheses of the PHU prepolymers were conducted following a modified literature procedure.^40^ The stoichiometric excess of BCC relative to TTDDA and the amounts of reagents used during the synthesis are presented in Table 1 and in the Supporting Information (Table S1). The BCC was placed in a 250 mL three-necked reactor equipped with a magnetic stirrer, thermometer, and an argon inlet. Next, the appropriate amount of TTDDA was added to the reactor. The synthesis was carried out at 60 °C for 24 h until the FT-IR and ^1^H NMR spectra of the sampled reactive mixture did not show further changes in the characteristic functional groups. The final prepolymers were obtained as yellowish, viscous, water-soluble liquids. Theoretical molar masses of the PRE_1.1 and PRE_1.2 were calculated based on eq S2 and equalled 8,500 and 4,500 g·mol^–1^, respectively. These values were used for stoichiometric calculations in further synthesis.

**Table 1:** Reaction Stoichiometry Used in the Synthesis of PHU Prepolymers, Obtained Contents of Functional Groups, and Molar Mass Data

		carbonate		urethane	*M̅*_n(NMR)_/	*M̅*_n_/	*M̅*_w_/	
		groups/mol %		groups/mol %	g·mol^–1^	g·mol^–1^	g·mol^–1^	*Đ*_M_/-
sample	BCC/TTDDA molar ratio/-	theo.^a^	calc.^b^	calc.^b^	calc.^b^	experimental^c^		
PRE_1.1	1.1: 1:0	9.15	10.00	18.50	7,600	7,800	23,300	2.98
PRE_1.2	1.2: 1:0	16.72	18.90	15.46	3,900	3,700	10,700	3.04

a Estimated theoretically from reaction stoichiometry (Table S1).

b Calculated based on the ^1^H NMR spectra (Figures S6 and S7) according to eqs S3–S6.

c Obtained from SEC measurements.

#### PRE_1.1

^1^H NMR (400 MHz, CDCl_3_): δ (ppm) = 1.71 (CH_2_CH_2_NH, 35.93 H), 2.76–2.99 (OH_I_, OH_II_, 24.62 H), 3.20 (CH_2_NH, 35.37 H), 3.49–3.69 (CH_2_O, 623.39 H), 3.92 (CH_2_OH, 27.32 H), 4.00 (CH_2_OC(O)NH, 33.73 H), 4.08 (CHOH, 30.90 H), 4.36 (CH_cyclic′_, 2.00 H), 4.45 (CH_cyclic″_, 2.00 H), 4.79 (NHC(O)O + CH_cyclic_, 11.62 H), 5.60 (CHOC(O)NH, 18.16 H). ^13^C NMR (100 MHz, DMSO-*d*^6^): δ (ppm) = 29.42 (CH_2_CH_2_NH), 38.92 (CH_2_NH), 62.12 (CH_2_OH), 66.15 (CH_2_OC(O)NH), 69.07 (CH_2cyclic_), 69.30 (CHOH), 70.10 (CH_2_O), 70.50 (OCH_2_CH_2_O), 72.38 (CHOC(O)NH), 75.10 (CH_cyclic_), 154.86 (C=O_cyclic_), 156.40 (HNC(O)O), 156.85 (HNC(O)O). FT-IR (ATR): 3343, 2870, 1796, 1708, 1453, 1245, 1092 cm^–1^.

#### PRE_1.2

^1^H NMR (400 MHz, CDCl_3_): δ (ppm) = 1.73 (CH_2_CH_2_NH, 17.18 H), 2.75 (OH_I_, 7.95 H), 3.06 (OH_II_, 1.37 H), 3.23 (CH_2_NH, 17.33 H), 3.50–3.71 (CH_2_O, 379.18 H), 3.94 (CH_2_OH, 10.92 H), 4.02 (CH_2_OC(O)NH, 9.56 H), 4.09 (CHOH, 11.97 H), 4.38 (CH_cyclic′_, 2.00 H), 4.47 (CH_cyclic″_, 2.00 H), 4.81 (NHC(O)O + CH_cyclic_, 5.22 H), 5.59 (CHOC(O)NH, 7.75 H). ^13^C NMR (100 MHz, DMSO-*d*^6^): δ (ppm) = 29.45 (CH_2_CH_2_NH), 38.99 (CH_2_NH), 62.18 (CH_2_OH), 66.18 (CH_2_OC(O)NH), 69.13 (CH_2cyclic_), 69.35 (CHOH), 70.14 (CH_2_O), 70.55 (OCH_2_CH_2_O), 72.43 (CHOC(O)NH), 75.13 (CH_cyclic_), 154.98 (C=O_cyclic_), 156.44 (HNC(O)O), 156.89 (HNC(O)O). FT-IR (ATR): 3342, 2866, 1799, 1712, 1456, 1254, 1092 cm^–1^.

### Reactive Extrusion (REX) Synthesis of Isocyanate-Free Hydrophobically Modified Ethoxylated Poly(hydroxy-urethane)s (IFHEURs)

IFHEURs were synthesized by chain extension of prepolymers using PRI. The applied molar ratio between the PRI and the prepolymers was in a range of 0.8–1.2:1.0, respectively. The detailed amounts of the reagents and the temperature applied during the reaction are listed in the Supporting Information (Table S2).

The REX synthesis of IFHEURs was carried out in a laboratory extruder Thermo Fisher Scientific HAAKE MiniLab II Micro Compounder using corotating conical twin screws (109.5 mm length, 14-5 mm diameter) with a conveying design (Figure S30). The extruder was equipped with a backflow channel and a control valve to switch between circulation and discharge of the processed material. The total capacity of the extruder was about 8 g, which allowed to obtain ca. 5 g of product. To assure the correct molar ratio between the reactants, the appropriate amounts of prepolymer and PRI were mixed in a PTFE beaker under argon for 5 min at 80 °C using a mechanical dissolver at 500 rpm, directly before the synthesis. Then, the premix was fed into the extruder, set to circulation mode. The synthesis was carried under a constant argon flow at 100 or 120 °C and a screw rotation speed of 100 rpm. The reaction progress was monitored through a change of the apparent melt viscosity (η_m_^*^), measured in the backflow channel, which functioned as an online viscometer according to the principle described in the Supporting Information (eqs S8–S10 and Table S3). Furthermore, samples of the reaction mixture (ca. 30 mg) were extracted from the backflow channel, accessed by removing a blind plug located above it and briefly stopping the screw rotation (Figure S30). The REX process was carried out for ca. 2 h until the melt viscosity plateaued and the FT-IR spectra of the sampled product did not show further changes in the characteristic functional groups. Then, the valve was opened toward the die channel and the product was discharged. The IFHEURs were obtained as yellowish, highly viscous, water-soluble liquids.

#### PRE_1.1_PRI(1.0)

^1^H NMR (400 MHz, CDCl_3_): δ (ppm) = 0.83 (CH_3_, 0.43 H), 1.20–1.43 (CH, CH_2_, 2.76 H), 1.73 (CH_2_CH_2_NH, 2.00 H), 2.48 (OH_I_, 0.04 H), 2.81 (CH_2_NH_2_, 0.11 H), 3.09 (OH_II_, 0.12 H), 3.22 (CH_2_NH, 2.41 H), 3.49–3.70 (CH_2_O, 27.29 H), 3.93 (CH_2_OH, 0.76 H), 4.01 (CH_2_OC(O)NH, 0.70 H), 4.09 (CHOH, 0.83 H), 4.37 (CH_cyclic′_, 0.02 H), 4.47 (CH_cyclic″_, 0.02 H), 4.80 (NHC(O)O + CH_cyclic_, 0.29 H), 5.60 (CHOC(O)NH, 0.67 H). ^13^C NMR (100 MHz, CDCl_3_): δ (ppm) = 14.16 (CH_3_), 18.01-26.81 (CH, CH_2_), 29.44 (CH_2_CH_2_NH), 38.93 (CH_2_NHC(O)O), 43.74 (CH_2_NH_2_), 62.05 (CH_2_OH), 66.17 (CH_2_OC(O)NH), 69.09 (CH_2cyclic_), 69.31 (CHOH), 70.13 (CH_2_O), 70.54 (OCH_2_CH_2_O), 72.41 (CHOC(O)NH), 75.21 (CH_cyclic_), 155.03 (C=O_cyclic_), 156.44 (HNC(O)O), 156.88 (HNC(O)O). FT-IR (ATR): 3344, 2920, 2869, 1797, 1704, 1455, 1350, 1092 cm^–1^.

#### PRE_1.2_PRI(1.0)

^1^H NMR (400 MHz, CDCl_3_): δ (ppm) = 0.84 (CH_3_, 0.97 H), 1.22-1.43 (CH, CH_2_, 6.83 H), 1.73 (CH_2_CH_2_NH, 2.00 H), 2.49 (OH_I_, 0.10 H), 2.79 (CH_2_NH_2_, 0.08 H), 3.10 (OH_II_, 0.27 H), 3.22 (CH_2_NH, 2.09 H), 3.52–3.70 (CH_2_O, 27.91 H), 3.94 (CH_2_OH, 0.77 H), 4.03 (CH_2_OC(O)NH, 0.74 H), 4.10 (CHOH, 0.86 H), 4.38 (CH_cyclic′_, 0.02 H), 4.47 (CH_cyclic″_, 0.02 H), 4.81 (NHC(O)O + CH_cyclic_, 0.33 H), 5.62 (CHOC(O)NH, 0.64 H). ^13^C NMR (100 MHz, CDCl_3_): δ (ppm) = 14.19 (CH_3_), 18.28-26.85 (CH, CH_2_), 29.48 (CH_2_CH_2_NH), 38.94 (CH_2_NHC(O)O), 43.69 (CH_2_NH_2_), 62.15 (CH_2_OH), 66.20 (CH_2_OC(O)NH), 69.14 (CH_2cyclic_), 69.34 (CHOH), 70.18 (CH_2_O), 70.58 (OCH_2_CH_2_O), 72.46 (CHOC(O)NH), 74.85 (CH_cyclic_), 155.09 (C=O_cyclic_), 156.46 (HNC(O)O), 156.92 (HNC(O)O). FT-IR (ATR): 3344, 2923, 2866, 1799, 1699, 1458, 1353, 1097 cm^–1^.

## Results and Discussion

### Synthesis and Structural Analysis of the Hydrophilic Poly(hydroxy-urethane) (PHU) Prepolymers

**Scheme 2:**
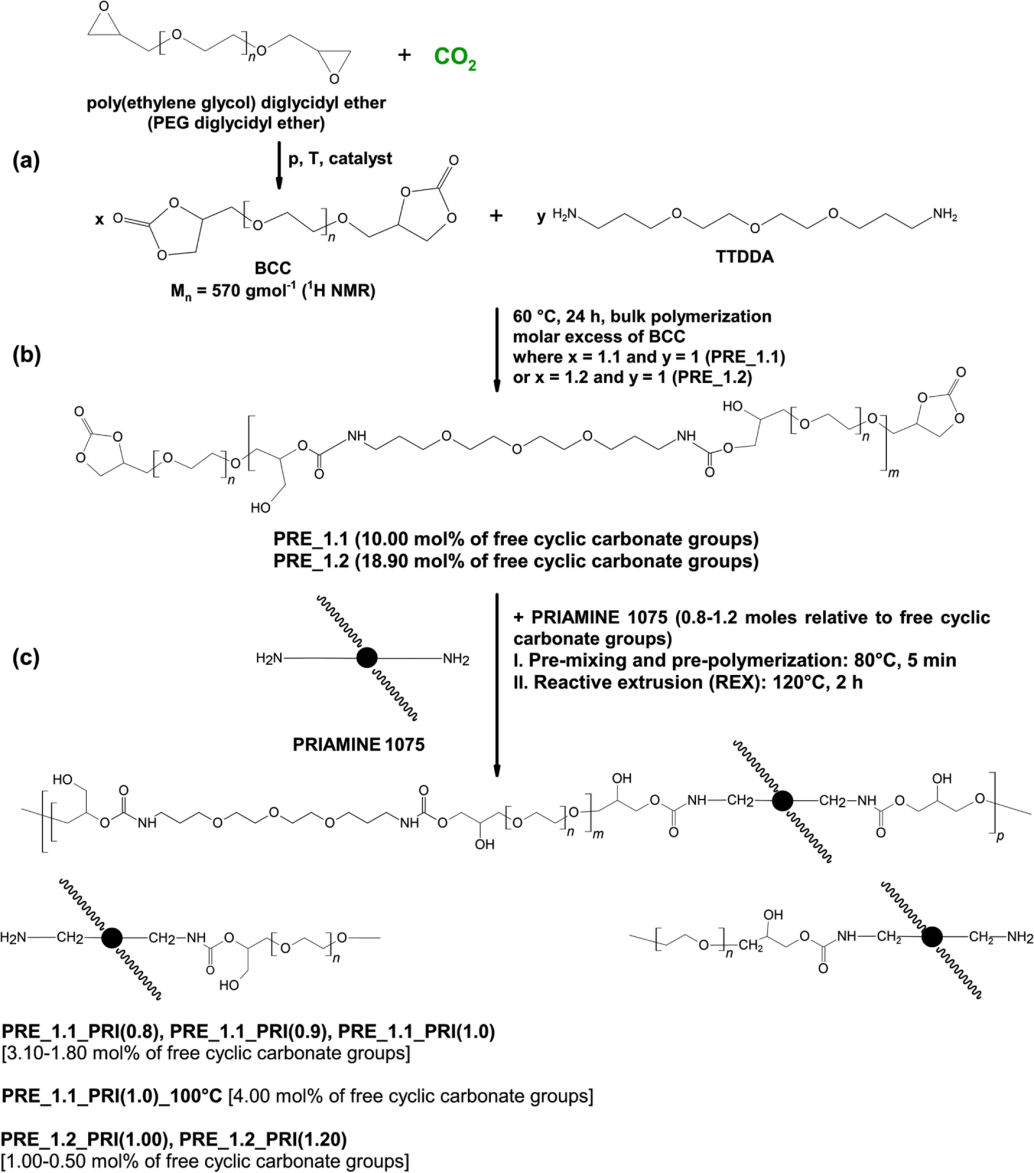
CO_2_-Based and “Green” Route toward (a) Cyclic Carbonate BCC Monomer, (b) PHU prepolymers, and (c) Isocyanate-Free Hydrophobically Modified Ethoxylated Poly(hydroxy-urethane) IFHEUR Thickeners

The monomeric precursor for the hydrophilic segment, poly(ethylene glycol) bis(cyclic carbonate) (BCC), was produced using CO_2_ as a “green” carbonation agent, as shown in Scheme 2a. Subsequently, the PHU prepolymers were synthesized through aminolysis of BCC using 4,7,10-trioxa-1,13-tridecanediamine (TTDDA) in solvent-free bulk polymerization. The synthesis was carried out with molar excess of BCC compared to TTDDA to obtain telechelic structures of the prepolymers with cyclic carbonate as reactive terminal groups. The names of the prepolymers indicate the applied molar ratio between BCC and TTDDA—1.1:1.0 for PRE_1.1 and 1.2:1.0 for PRE_1.2 (Table 1 and Scheme 2b). The resulting chain length and content of functional groups varied between the prepolymers, depending on the stoichiometry of the formulation. The structure of the prepolymers was investigated using FT-IR, NMR, MALDI-ToF, and SEC analyses (Table 1, Figures S4–S11 and 2).

**Figure 2:**
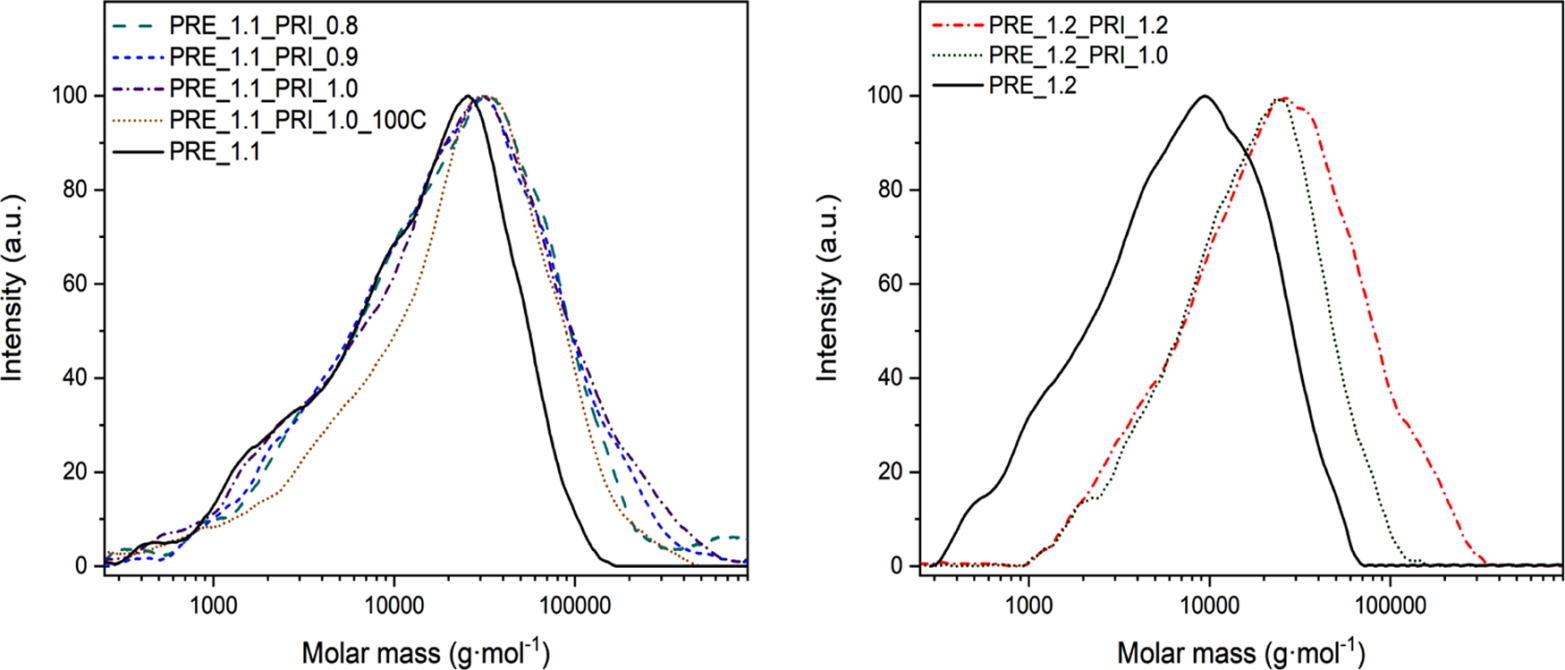
Molar mass distribution of IFHEURs based on PRE_1.1 (left) and PRE_1.2 (right).

The FT-IR spectra confirmed the ring-opening aminolysis of BCC (Figures S4 and S5). The intensity of the absorption band of the cyclic carbonate groups at about 1799 cm^–1^ decreased with the progress of the reaction between BCC and TTDDA; however, the band remained distinct in the final prepolymers. The absorption bands corresponding to the stretching vibrations of C=O in urethane groups were observed at 1708–1712 cm^–1^. Other bands visible in spectra at about 1533–1536 cm^–1^ (sharp) and 3343 cm^–1^ (broad) were assigned to deforming vibrations of N–H bonds in the urethane and H-bonded O–H groups, respectively. The CH_2_ and CH groups gave rise to a broad absorption band at about 2866–2970 cm^–1^.

The formation of bonds typical for hydroxy-urethanes and incorporation of the cyclic carbonate end groups in the obtained prepolymers were proven with ^1^H NMR spectroscopy (Figures S6 and S7). The presence of the free cyclic carbonates was confirmed by the resonances at 4.38 and 4.47 ppm, ascribed to the cyclic CH_2_ groups. Their decrease in intensity was monitored along the synthesis and allowed to determine when the maximal conversion of the carbonate groups was reached. The characteristic signal of the urethane proton (NHC(O)O) was observed in the prepolymers at about 4.80 ppm. The signals corresponding to CH and CH_2_ groups neighboring −OC(O)NH groups were detected at 5.60 and 4.00 ppm, respectively. Furthermore, the signals at about 4.08 ppm and 3.92 ppm were ascribed to the CH and CH_2_ groups adjacent to O–H groups, respectively. These signals (5.60, 4.00, 3.92, and 4.08 ppm) indicated formation of two hydroxy-urethane isomers (1,2-isomer and 1,3-isomer) during the aminolysis of BCC.^41^ The group of signals within the range of 3.50–3.71 ppm was attributed to CH groups neighboring the oxygen atoms in oxyethylene moieties.

^13^C NMR spectroscopy verified the formation of hydroxy-urethane moieties in the prepolymers (Figures S8 and S9). The signals from two chemically inequivalent carbonyl carbon atoms in the urethane groups, arising from the 1,2-isomer or 1,3-isomer units, were observed at 156.85 and 156.42 ppm, respectively. The signals at 72.40 and 66.16 ppm belonged to carbon atoms from CH and CH_2_ groups adjacent to −OC(O)NH, while the signals from the same carbon atoms neighboring the O–H group were visible at 69.31 and 62.15 ppm, respectively. The signal of the carbon atom from CH_2_ groups adjacent to −NHC(O)O groups appeared in spectra at about 38.95 ppm. The presence of ether groups in the prepolymers was evidenced by the signals between 70.12 and 70.52 ppm. Additionally, the signal of carbonyl carbon atoms from the cyclic carbonate groups was detected at 154.92 ppm.

FT-IR spectroscopy indicated that PRE_1.1 and PRE_1.2 did not contain any urea bonds due to the lack of absorption bands at about 1645 and 1676 cm^–1^.^42^ Similarly, the characteristic signals corresponding to the urea moieties in ^1^H NMR at about 5.76 ppm^43^ and in ^13^C NMR at about 158.58 ppm^43^ were not observed in the spectra. It was thus confirmed that urea byproducts were not formed during the synthesis of the prepolymers.

The ^1^H NMR spectra of prepolymers were used to calculate the contents of terminal cyclic carbonate groups and urethane groups formed after the synthesis and estimate the number-average molar mass (*M̅*_n(NMR)_) of the obtained species. The data are listed in Table 1, and the calculation method is shown in the Supporting Information (Figures S6 and S7, eqs S3–S6). As expected, the calculated amount of free cyclic carbonates was higher in the case of PRE_1.2, synthesized using a larger molar excess of BCC, of ca. 18.90 mol %, compared to PRE_1.1 containing ca. 10.00 mol %. The values obtained based on the ^1^H NMR evaluation were lower relative to the theoretical estimation from the reaction stoichiometry, which at full conversion should yield 16.72 and 9.15 mol % for PRE_1.2 and PRE_1.1, respectively. The differences between the calculated and theoretical values can be ascribed to incomplete conversion of the cyclic carbonate groups during the aminolysis of BCC, which is commonly known for PHU polymerization.^32^ Furthermore, PRE_1.1 showed a noticeably higher content of urethane groups and almost doubled *M̅*_n(NMR)_ compared to PRE_1.2. The *M̅*_n(NMR)_ values of 7,600 and 3,900 g·mol^–1^ calculated for PRE_1.1 and PRE_1.2, respectively, were consistent with the corresponding number-average molar mass (*M̅*_n_) obtained experimentally from SEC analyses (Table 1 and Figure 2). Moreover, both prepolymers exhibited similar dispersity (*Đ*_M_) of approx. 3.00, while their weight-average molar mass (*M̅*_w_) reached ca. 23,300 g·mol^–1^ for PRE_1.1 and ca. 10,700 g·mol^–1^ for PRE_1.2.

**Scheme 3:**
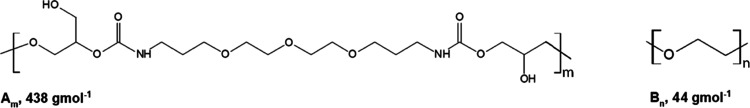
Structure and Molar Mass of the Repeating Units in the Backbone Detected for Prepolymers in the MALDI-ToF Spectra

**Scheme 4:**
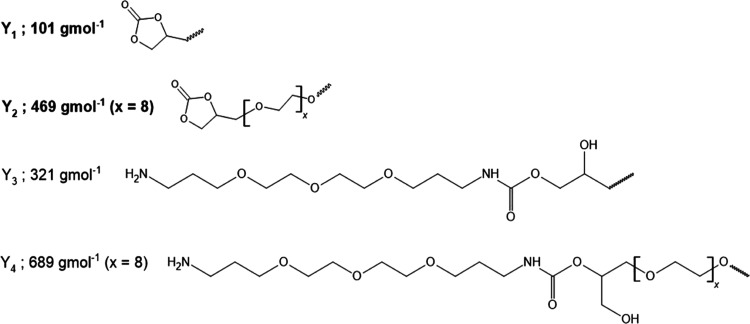
Structure and Molar mass of the Terminal Groups Detected for Prepolymers in the MALDI-ToF Spectra

MALDI-ToF mass spectrometry was used to further delve into the structural analysis of the obtained PHU prepolymers and determine the type of repeating units in the backbone and the terminal groups. The selected fragments of the MALDI-ToF mass spectra are presented in Figures S10 and S11. Due to formation of diverse structures in the prepolymer chains, the spectra showed groups of signals instead of single signals. The detected architecture of the repeating units in the core and the end groups of the prepolymers, along with their molar mass are shown in Schemes 3 and 4, respectively.

The major fraction of both PRE_1.1 and PRE_1.2 contained the ethoxylated repeating units A*_m_* and B*_n_* (Scheme 3), as well as cyclic carbonate end groups Y_1_ and Y_2_ (Scheme 4). This confirmed the formation of the desired bifunctional telechelic PHU prepolymers. Smaller populations of macromolecules terminated with one cyclic carbonate group (Y_1_/Y_2_) and one amine group (Y_4_/Y_3_) were also observed in the mass spectra. As formulations of the prepolymers were modified by changing the molar excess between the BCC and TTDDA, the obtained structures varied mainly in the distance between the terminal cyclic carbonates in the hydrophilic segment. The MALDI-ToF analysis confirmed that PRE_1.2 contained higher content of cyclic carbonate end groups Y_1_, and Y_2_ compared to PRE_1.1. The *m*/*z* values of the signal series marked in Figures S10 and S11 were corresponding to the total molar masses of the end groups Y_1_, Y_2_, Y_3_, and/or Y_4_, (Scheme 4), repeating units A*_m_* and B*_n_* (Scheme 3), and potassium cation (39 g·mol^–1^). The examples are as follows: A_2_ B_6_ (Y_1_, Y_2_), K^+^ (1749 *m*/*z*), A_3_ B_6_ (Y_1_, Y_2_), K^+^ (2187 *m*/*z*), A_3_ B_7_ (Y_1_, Y_2_), K^+^ (2231 *m*/*z*). The differences in *m*/*z* values of the A_2_ B_6_ (Y_1_, Y_2_), K^+^ and A_3_ B_6_ (Y_1_, Y_2_), K^+^, as well as A_3_ B_6_ (Y_1_, Y_2_), K^+^ and A_3_ B_7_ (Y_1_, Y_2_), K^+^ signals, equal 438 and 44 *m*/*z*, respectively.

### Reactive Extrusion (REX) Synthesis and Structural Analysis of the Isocyanate-Free Hydrophobically Modified Ethoxylated Poly(hydroxy-urethane)s (IFHEURs)

Hydrophobic modification of IFHEUR was achieved by copolymerizing the hydrophilic PHU prepolymers with the difunctional PRI, containing highly hydrophobic, pendant aliphatic chains (Schemes 1 and 2c). The obtained IFHEURs with varied molar ratios between the PRI and the selected prepolymer are listed in Table 2, while the formulation details are shown in Table S2. The names of the obtained IFHEURs (e.g., PRE_1.2_PRI(0.8)) indicate the type of prepolymer used (e.g., PRE_1.2) and the molar ratio between PRI and the prepolymer (e.g., 0.8). The reaction stoichiometry was adjusted based on the chain length of the applied prepolymer to reach a sufficiently high molar mass and a varied number of hydrophobic PRI moieties built into a single IFHEUR molecule.

**Table 2:** Reaction Stoichiometry and Content of Functional Groups in the Obtained IFHEURs, along with the Molar Mass Data Obtained from SEC and the Final Viscosity of the Melt Measured Online during REX Synthesis

		carbonate		urethane	amine	*M̅*_w_/		
		groups/mol %		groups/mol %	groups/mol %	g·mol^–1^	*Đ*_M_/-	η_m_^*^^c^/Pa·s
sample	PRE/PRI molar ratio /-	theo.^a^	calc.^b^	calc.^b^	calc.^b^	experimental		
PRE_1.1_PRI(0.8)	1.0: 0.8	2.45	3.10	22.50	1.04	36,500	3.40	30
PRE_1.1_PRI(0.9)	1.0: 0.9	1.24	2.40	23.89	1.35	38,900	4.08	34
PRE_1.1_PRI(1.0)	1.0: 1.0	0.00	1.80	24.20	1.44	46,300	4.67	36
PRE_1.1_PRI(1.0)_100C	1.0: 1.0	0.00	4.00	22.92	1.70	39,600	2.74	41
PRE_1.2_PRI(1.0)	1.0: 1.0	0.00	1.00	25.34	1.24	23,900	2.46	16
PRE_1.2_PRI(1.2)	1.0: 1.2	0.00	0.50	25.93	2.07	39,700	3.42	30

a Estimated theoretically from reaction stoichiometry (Table S2).

b Calculated based on the ^1^H NMR spectra (Figures S18–S23) according to eqs S5–S7.

c At 100 °C for PRE_1.1_PRI(1.0)_100C and 120 °C for other IFHEURs (Table S3).

The syntheses were carried out using solvent-free REX, suitable for mass polymerization of the highly viscous mixture. As previously mentioned, the course of the reaction was monitored through online rheological measurements carried out in the extruder (Supporting Information, eqs S8–S10) and offline FT-IR analysis, performed on samples collected during the REX process. FT-IR spectroscopy allowed us to track the structural changes in the IFHEUR products. The spectra showed that the intensity of the absorption band at 1799 cm^–1^ declined along the synthesis, which indicated progressing conversion of the cyclic carbonate groups. The aminolysis was further confirmed through the increasing intensity of bands at about 3344 and 1700 cm^–1^, corresponding to H-bonded O–H groups and to the stretching vibrations of C=O in urethane groups, respectively, while urea byproducts at 1645 and 1676 cm^–1^ were not detected.^8^ Furthermore, the broad absorption band of the aliphatic groups, previously observed in prepolymers between 2866 and 2970 cm^–1^, was divided in the IFHEUR spectra into two absorption bands at about 2866 and 2923 cm^–1^ due to the presence of CH_3_ and CH_2_ groups in the PRI moieties. It was considered that the maximum degree of chain extension in the IFHEURs was reached after ca. 2 h of REX, once the detected cyclic carbonate absorption band did not show a further decrease of intensity (Figures S12–S17). As bulk syntheses of PHUs, carried out in conventional batch reactors, typically require significantly longer reaction times to reach sufficient conversion, the faster reaction in the extruder can be attributed to its superior mixing ability. It is expected that the REX technique promoted monomer diffusion despite the high viscosity of the reactants, increasing substantially with rising conversion, and therefore shortened the required synthesis time.

The rheological measurements carried out online during the REX process confirmed the increase of melt viscosity, corresponding to the growth of polymeric chains. The viscosity curves showed a plateau at elevated conversion, while the apparent melt viscosity (η_m_^*^), recorded at the end of the synthesis at a given temperature, indicated a varied degree of chain extension between the IFHEURs (Tables 2 and S3). These trends could be ascribed to the structural properties of the obtained specimens. To delve into the molecular architecture and hydrophobic functionalization, crucial for associative performance, the obtained IFHEURs were investigated using NMR and SEC analyses (Table 2 and Figures 2 and S12–S29).

**Figure 3:**
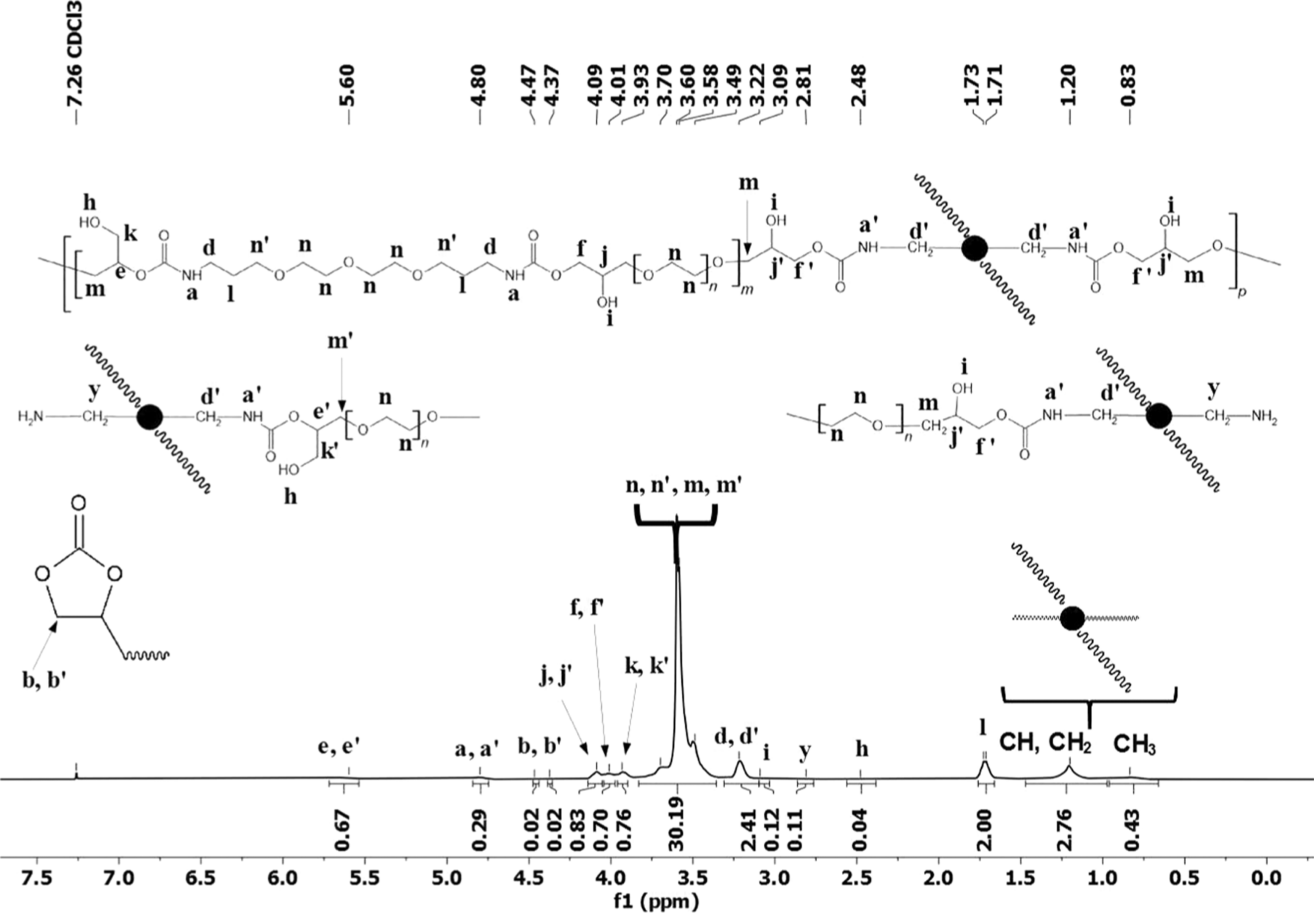
^1^H NMR spectra of PRE_1.1_PRI(1.0).

NMR spectroscopy allowed to elucidate the structures of the final IFHEUR products. The obtained spectra are shown in the Supporting Information (Figures S18–S29), while an exemplary ^1^H NMR spectrum of PRE_1.1_PRI(1.0) is presented in Figure 3. Both ^1^H and ^13^C NMR spectra showed signals analogues to the prepolymers (Figures S6–S9) incorporated as hydrophilic segments in the respective IFHEURs. However, the intensity of signals in the ^1^H NMR spectra corresponding to the free cyclic carbonate at 4.37 and 4.47 ppm was detectably reduced. The signals dd′, corresponding to the CH_2_ group present in both hydrophilic and hydrophobic segments, neighboring the urethane group in an α-position, were detected at 3.22 ppm. The CH_2_ groups, adjoining the urethane groups in a β-position only in the hydrophilic chains, were ascribed to the signal l at 1.73 ppm. As the integral intensity ratio between the signals dd′ relative to signal l increased, it indicated formation of new urethane bonds and confirmed chain extension of the prepolymers with PRI moieties. The ^1^H NMR spectra also revealed new signals ascribed to the alkyl chains present in the backbone and pendant groups of PRI—CH and CH_2_ groups at 1.20 ppm and CH_3_ groups at 0.83 ppm. The signal from the CH_2_ groups, adjacent to the amine group in a free end chain of PRI, was detected at 2.81 ppm. The corresponding new signals from PRI were also detected in the ^13^C NMR spectra—CH_3_, CH_2_, and CH groups at about 14.16 and 18.01–26.81 ppm and CH_2_NH_2_ at 43.74 ppm. As neither ^1^H nor ^13^C NMR spectra showed the signals corresponding to urea bonds at 5.76^43^ and 158.58 ppm,^43^ respectively, it can be stated that the side reaction did not occur during the applied synthesis procedure. Thus, the NMR confirmed formation of the desired hydroxy-urethane structure in the IFHEURs and verified the observation from FT-IR analysis, concerning the purity of the obtained products. The studies concerning bulk polymerization of PHU typically report that the amounts of urea byproducts formed at a temperature up to 120 °C are moderated; however, their complete absence is uncommon.^44^ It can be therefore expected that the effective mixing and heat distribution in the extruder, improved in comparison to conventional batch reactors, prevents local overheating of the melt and impedes the thermally induced side reactions.

The content of functional groups present in the IFHEURs was estimated based on the ^1^H NMR spectra to gain insight into the architectural differences in the obtained species (Figures S18–S23, eqs S5–S7). The calculated concentrations of the remaining cyclic carbonate and amine groups and the newly formed urethane groups are summarized in Table 2. The consumption of the reactive groups was significantly increased when the synthesis temperature was increased from 100 to 120 °C for the stoichiometric formulations in PRE_1.1_PRI(1.0)_100C and PRE_1.1_PRI(1.0), respectively. Thus, the REX temperature of 120 °C, applied for the study of IFHEUR formulations, assured optimal conversion without the risk of side reactions. It is expected that high temperatures could promote faster reactions and further chain extension but also lead to thermally induced urea formation.

The gradual increase of molar ratio between the PRI and prepolymers in the IFHEURs synthesized at 120 °C led to correspondingly increased conversion of the cyclic carbonate groups. At stoichiometric amounts of the reactants, the concentrations of free cyclic carbonates were reduced to ca. 1.80 mol % for PRE_1.1_PRI(1.0) and 1.00 mol % for PRE_1.2_PRI(1.0). The higher conversion in the case of IFHEUR based on the shorter prepolymer PRE_1.2 could be related to its moderate η_m_^*^ of ca. 16 Pa·s. It is expected that the less viscous melt facilitated better mobility of the reactive species compared to the mixture containing the longer PRE_1.1, for which the η_m_^*^ was more than doubled (ca. 36 Pa·s). The detected content of the remaining carbonate groups was above the value calculated theoretically for full conversion at the applied molar ratio between the reactants. This pointed toward a similar mechanism limiting the reactivity of cyclic carbonates, as previously observed during prepolymer synthesis. It should be however noted that this effect was not exacerbated despite the reduced diffusion of reactive species, which typically occurs at high conversions during step-growth polyaddition and is especially severe in the case of PHU systems. Furthermore, using moderate excess of amine groups in the formulation of PRE_1.2_PRI(1.2) allowed further chain extension and reduction of the carbonate groups down to 0.50 mol %. A residual amount of cyclic carbonates was detected in all of the obtained samples, which indicated that the hydrophilic segments from the prepolymers were present as terminal chains in the IFHEURs.

The attachment of PRI molecules into the prepolymers was confirmed by the increased content of urethane groups in the IFHEURs, compared to the starting prepolymers. The detected amount of the newly formed urethane bonds in the obtained formulations corresponded to the previously observed trend in the conversion of the cyclic carbonates. The concentration of the urethane groups increased from ca. 18.50 to 24.20 mol % for PRE_1.1_PR(1.0), obtained by chain extension of PRE_1.1 at a stoichiometric ratio of reactants. In the case of analogous synthesis with the use of PRE_1.2, containing more free cyclic carbonate groups, the amount of the urethane bonds increased from 15.46 to 25.34 mol % in the resulting PRE_1.2_PR(1.0). The further reaction was enabled by a moderate molar excess of PRI in the PRE_1.2_PRI(1.2) as its content of urethane groups was raised to ca. 25.93 mol %. Thus, it can be assumed that the number of hydrophobic groups, located in the core of the IFHEUR molecules due to chain extension of prepolymers with PRI, varies dependent on the applied reaction stoichiometry. Since the concentration of free amine groups detected in the IFHEURs showed a corresponding increase at higher content of PRI, it confirmed that a significant fraction of the species was also end-capped with the hydrophobic groups.

The extent of chain growth, and therefore the trend in the quantity of the hydrophobic centers incorporated in the core and as terminal groups of IFHEURs, could be estimated based on SEC analysis. The data on the molar mass distribution and molar mass average obtained from SEC for the IFHEURs are given in Table 2, while the chromatograms are presented in Figure 2. Depending on the formulation, the *M̅*_w_ of the IFHEURs based on the shorter and longer prepolymer varied between 23,800–39,700 and 33,200–46,300 g·mol^–1^, respectively. Higher *M̅*_w_ and *Đ*_M_ were obtained with increased length of the prepolymer and molar ratio between PRI and the prepolymer. The same holds true when the temperature of the synthesis was increased from 100 to 120 °C. As expected, the trend between the reached molar mass averages of the IFHEUR species correlated to the η_m_^*^, previously observed during REX synthesis.

All of the obtained IFHEURs showed a monomodal distribution of molar mass with only a slight shift of the main peak in the range of 25,000–30,000 g·mol^–1^. The observed increase in *M̅*_w_ and *Đ*_M_ at higher content of PRI was explained by the formation of a small fraction of significantly extended chains. Large molecules were produced in the case of the IFHEURs synthesized from the longer prepolymer PRE_1.1 at 120 °C when the molar ratio between the PRI and prepolymer was increased above 0.8. The fraction with high molar mass was visible in the chromatograms of PRE_1.1_PRI(0.9) and PRE_1.1_PRI(1.0) as a shoulder between 150,000–300,000 g·mol^–1^ and 180,000–500,000 g·mol^–1^, respectively. These long-chain species were not obtained during the synthesis of PRE_1.1_PRI(1.0)_100C at 100 °C. In this specimen, the increase of *M̅*_w_ and the decrease of *Đ*_M_ were due to the broadening of the main peak above 30,000 g·mol^–1^ and conversion of the low molar mass fraction containing shorter prepolymer chains. Thus, it is expected that the hydrophobes are differently distributed along the molecules of PRE_1.1_PRI(1.0)_100C compared to the IFHEURs obtained at 120 °C.

The elevated content of PRI allowed the formation of large IFHEUR molecules using shorter prepolymer PRE_1.2 at 120 °C. The high molar mass shoulder was observed in the chromatogram of PRE_1.2_PRI(1.2) between 130,000 and 300,000 g·mol^–1^, i.e., in a similar range as in the case of PRE_1.1_PRI(0.9). As these samples had comparable molar mass distribution, their structures differed mainly in the number of the built-in hydrophobic groups from PRI and their spacing between the hydrophilic prepolymer segments. In general, it can be stated that the IFHEURs with longer chains contained higher fractions of the hydrophobic centers, located in the core of the molecule and incorporated as end groups.

### Steady Shear Behavior of IFHEURs in Aqueous Solutions

**Figure 4:**
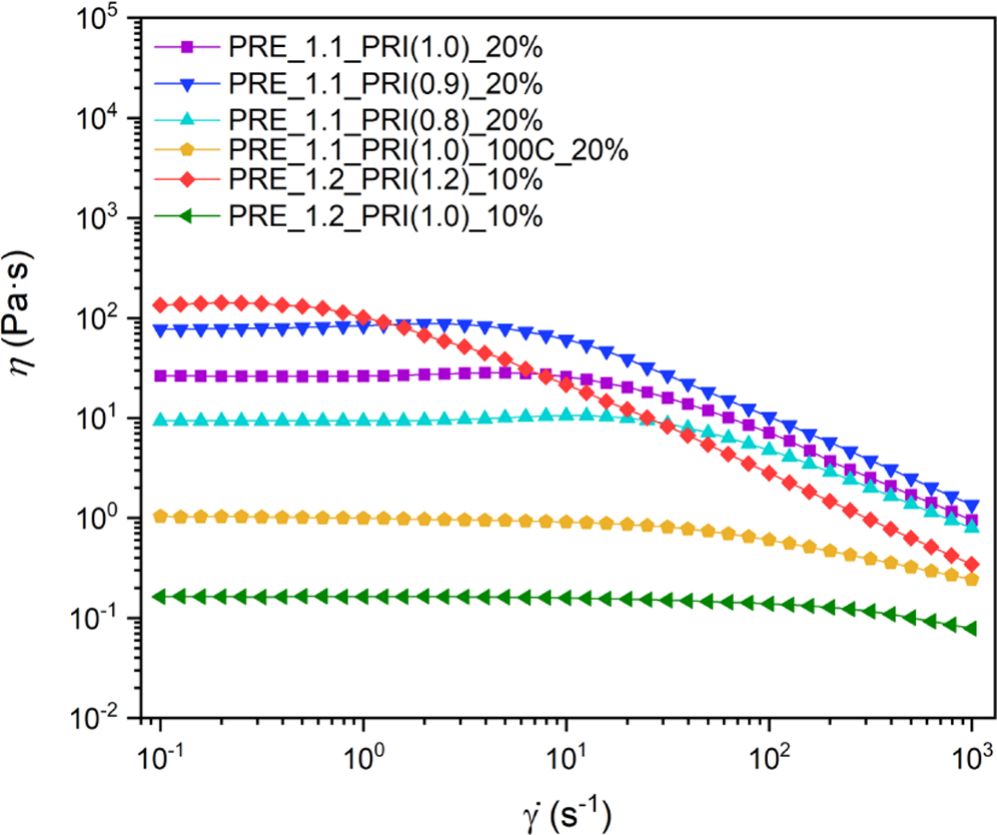
Plots of steady shear viscosity (η) in dependence on the shear rate (γ̇) for the aqueous solutions of IFHEUR at the indicated concentration of 10 or 20 wt %.

The tailored chain length of the hydrophilic segments and the number of the built-in hydrophobic centers yielded a series of IFHEURs with distinct differences in the molecular architecture. Their rheological behavior in aqueous solutions was investigated to correlate the associative mechanism to the structural differences between the species. The profiles of steady shear viscosity (η) as a function of the shear rate (γ̇) for aqueous solutions of the IFHEURs at indicated concentrations are shown in Figure 4. The IFHEUR series obtained from longer prepolymer PRE_1.1 were tested at a concentration of 20 wt %. IFHEURs based on PRE_1.2 were investigated after dilution to 10 wt %, as the 20 wt % solutions were too viscous to carry out a rotational measurement in the available setup of the rheometer. The performance of IFHEURs containing the hydrophilic segments from PRE_1.1 or PRE_1.2 cannot be directly compared due to the different concentrations of the used solutions. However, general conclusions regarding the influence of their architecture on the rheological behavior can be drawn since the studied IFHEURs have the sample type of the PRI hydrophobe. These structural factors include molar mass, molar mass distribution, the number of hydrophobic centers, and the ratio between the length of the hydrophilic and hydrophobic chains.

As previously described, the increase in *M̅*_w_ and *Đ*_M_ obtained for IFHEURs with higher contents of hydrophobes was due to the formation of a small fraction of high molar mass species (Figure 2 and Table 2). This chain extension induced a considerable increase of the solution η in the case of specimens PRE_1.2_PRI(1.2), PRE_1.1_PRI(0.9), and PRE_1.1_PRI(1.0). As previously introduced, the three-dimensional network of the telechelic HEUR is based on micellelike junctions between the molecules. Intermolecular associations are formed when the hydrophilic backbone of the HEUR molecule bridges two neighboring micelles, each incorporating one of the end-capping hydrophobes. Therefore, it is expected that the large IFHEUR species, which carry multiple hydrophobic chains in a single molecule, strongly contribute to the formation of the network. Consequently, a lower *M̅*_w_ and a lack of the high molar mass fraction in the case of PRE_1.1_PRI(0.8) and PRE_1.1_PRI(1.0)_100C can be ascribed to their inferior thickening performance. Although the solutions of PRE_1.2_PRI(1.0) and PRE_1.2_PRI(1.2) were studied at lower concentrations compared to the IFHEURs based on the PRE_1.1, they are expected to exhibit similar tendencies between associative behavior and molar mass characteristics. In general, the structure–property studies of telechelic HEURs with multitail hydrophobes indicate that an increased number of the end-capper leads to a stronger association in aqueous solutions.^10,11^ However, data concerning HEURs with pendant hydrophobic chains, built-in as an chain extender within the core of the molecule, are limited. In the case of the investigated IFHEURs, the hydrophobic center can be located within the copolymer chain, as well as terminating it, while the number of the built-in hydrophobes increases with the molar mass of the species. Therefore, the impact of the molecular architecture on the associative mechanism of the new IFHEURs is expected to be more complex than in the conventional HEURs. A variety of interactions can potentially take place if two or more hydrophobic centers are built into an IFHEUR molecule. The hydrophobes, which are separated by a single hydrophilic segment, could undergo intramolecular aggregation into isolated micelles and create intermolecular connections between the neighboring junctions. Longer molecules, containing numerous hydrophobic centers spaced apart at a greater distance, could additionally link several micelles and form a superbridge.^28^

As indicated above, the rheological behavior of IFHEURs synthesized from the longer prepolymer PRE_1.1 was studied in an aqueous solution at a concentration of 20 wt %. The low solution η of the PRE_1.1_PRI(1.0)_100C pointed toward a predominant assembly into separated micelles, while the connections between the micellar junctions were scarce. It cannot be ruled out that the obtained IFHEURs contained a substantial fraction of molecules with a single hydrophobic group, formed by binding two PRE_1.1 with PRI. As formation of the network requires the presence of a minimum of two hydrophobes in a chain, such species cannot contribute to the mechanism of associative thickening. Furthermore, it is expected that the distribution of the hydrophobes in PRE_1.1_PRI(1.0)_100C varied compared to the IFHEUR series obtained at 120 °C due to the previously observed differences between their molar mass distribution. Thus, the intramolecular associations were promoted if certain fragments of the PRE_1.1_PRI(1.0)_100C molecules contained too short hydrophilic segments. The moderate solution η of PRE_1.1_PRI(0.8) indicated that a small number of the bridged junctions were present. In this case, the intramolecular association into separated micelles still played a considerable role, resulting in a sparse network. However, the increased molar mass and number of hydrophobic centers in PRE_1.1_PRI(0.9) and PRE_1.1_PRI(1.0) facilitated linking of the micellar junctions, led to a denser network, and, consequently, caused a significant increase of the solution η compared to PRE_1.1_PRI(0.8). As PRE_1.1_PRI(0.9) exhibited a greater value of η than PRE_1.1_PRI(1.0), despite having lower molar mass, it is plausible that interactions of the long-chain molecules had a dominating effect. As previously mentioned, the fraction of PRE_1.1_PRI(0.9) with high molar mass was in the range of 160,000–300,000 g·mol^–1^, while in the case of PRE_1.1_PRI(1.0), it reached above 500,000 g·mol^–1^. It can be assumed that the chains with molar mass in the range of PRE_1.1_PRI(0.9) unfolded into the solution, enabling expanded intermicellar connections and high association degrees. Furthermore, the formation of superbridges^28^ extended across the network could be expected. On the contrary, the reduced mobility of larger species in PRE_1.1_PRI(1.0) possibly limited the area of interactions, promoting local bridging of micellar junctions or intramolecular assembly of the highly hydrophobic chains.

As previously mentioned, solutions of IFHEURs containing the shorter hydrophilic segment from PRE_1.2 were studied at a concentration of 10 wt %. Thus, the dilution effect must be considered when interpreting the association effect in comparison to the 20 wt % solutions of IFHEURs obtained from PRE_1.1. Due to the lower molar mass of the PRE_1.2 prepolymer, the ratio between the length of the hydrophobic and hydrophilic components in resulting IFHEURs was higher relative to IFHEURs containing the longer prepolymer segment (Table 2). Therefore, at sufficient concentrations, PRE_1.2_PRI(1.0) and PRE_1.2_PRI(1.2) could form networks with shorter hydrophilic bridges between the micellar junctions and, consequently, a denser network. This phenomenon could be ascribed to the higher solution η reached by PRE_1.2_PRI(1.2) at 10 wt % compared to PRE_1.1_PRI(0.9) at 20 wt %, despite their similar fraction of high molar mass species at ca. 130,000–300,000 g·mol^–1^. It is expected that in the solution of PRE_1.2_PRI(1.0), diluted to 10 wt %, the distance between the neighboring micelles is too large for the majority of the molecules to form intermolecular connections. Thus, they undergo self-assembly into separated micelles, causing a significant decrease of η compared to PRE_1.2_PRI(1.2). Consequently, the results point toward the existence of an optimal range of molar mass, in which the obtained IFHEURs contribute to the formation of a well-interconnected network.

Furthermore, the shear thinning of the IFHEUR solutions can be attributed to the associative structure of their network and its susceptibility to disintegrate under shear stress. In principle, the hydrophobic tails can associate and dissociate from the micelles when the HEUR chains are relaxed in the aqueous media. Therefore, the micellar junctions are dynamically disengaged and reconnected in a finite time. The rate of the micellar dissociation/association corresponds to the relaxation behavior of the HEUR species. The connections are destroyed under shear stress if the hydrophobes are pulled out of the micellar junctions at a γ̇ higher than the inverse of the relaxation time of the network. In such a case, the removed chain reassembles into a single micelle and the η of the solution decreases. The networks with dominating intermolecular associative mechanisms exhibit stronger shear-induced reduction of η as the relaxation time increases with the association degree. Therefore, the solutions of scarcely interconnected PRE_1.2_PRI(1.0), at 10 wt %, and PRE_1.1_PRI(1.0)_100C, at 20 wt %, behaved as Newtonian fluids in a wide range of γ̇ and a slight shear thinning was noticeable only at high γ̇. The Newtonian plateau was reduced with increasing η, between the 20 wt % solutions of PRE_1.1_PRI(0.8), PRE_1.1_PRI(1.0), and PRE_1.1_PRI(0.9), while shear thinning occurred at lower γ̇ and became more pronounced. This could be attributed to a gradual formation of more complete networks, resulting in increased relaxation times. Furthermore, these specimens showed a slight shear thickening shortly before the shear-induced decrease of solution η. Similar to the telechelic HEURs, this could be related to stretching of the IFHEUR chains under shear, which facilitated rearrangement from separated micelles towards intermolecular connections. It can be assumed that the shear thickening was not observed in the case of the 10 wt % solution of PRE_1.2_PRI(1.2), which had the highest η among all of the investigated IFHEURs, due to the pre-existing extensively cross-linked structure. Indeed, the PRE_1.2_PRI(1.2) solution showed Newtonian behavior only in a narrow range of γ̇, followed by the strongest shear thinning effect. This indicates a highly developed physical network with a long relaxation time, which is expected to be further increased if a higher concentration of PRE_1.2_PRI(1.2) would be applied. As both PRE_1.1_PRI(0.9) and PRE_1.2_PRI(1.2) have comparable molar mass characteristics, a larger fraction of hydrophobic centers in the latter can be ascribed to reaching a higher association degree in the network. Therefore, the observed trend in the network strength points toward the influence of the number of built-in hydrophobic groups and the length of the IFHEUR molecules.

### Oscillatory Shear Measurements of IFHEUR Aqueous Solutions

**Figure 5:**
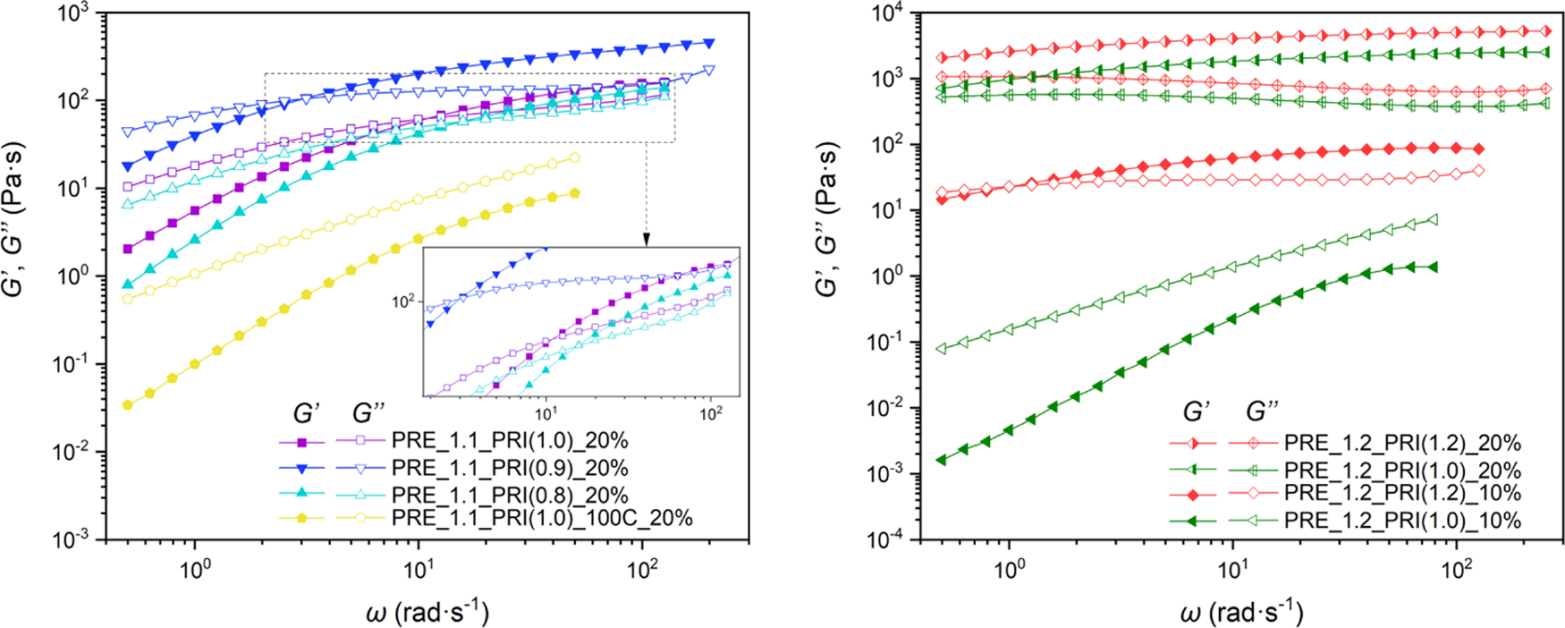
Plots of the storage (*G*′) and loss (*G*″) moduli in dependence o the angular frequency (ω) for the aqueous solutions of IFHEUR at the indicated concentration of 10 or 20 wt %.

To deepen the insight into the association mechanism indicated by the steady shear measurements, the oscillatory behavior was studied at equivalent concentrations: 10 wt % in the case of IFHEURs synthesized from PRE_1.2 and 20 wt % in the case of IFHEURs from PRE_1.1. The IFHEURs containing PRE_1.2 segments were additionally tested at a concentration of 20 wt % to verify the difference in the thickening performance relative to the IFHEURs with the hydrophilic spacer based on PRE_1.1. The storage (*G*′) and loss (*G*″) moduli as a function of the angular frequency (ω), obtained in the oscillatory shear measurements of the IFHEUR aqueous solutions, are presented in Figure 5.

The IFHEURs, containing the longer hydrophilic segment from PRE_1.1, showed an increase of both *G*′ and *G*″ over the whole ω range, while the change of slope was dependent on the associative structures present in the solution (Figure 5, right). In the case of the sample PRE_1.1_PRI(1.0)_100C, the *G*″ remained higher than the *G*′ independently of the applied ω. This pointed toward evident Newtonian fluid behavior due to the predominant intramolecular associations into isolated micelles. Specimens PRE_1.1_PRI(0.8), PRE_1.1_PRI(0.9), and PRE_1.1_PRI(1.0), containing a gradually increasing fraction of high molar mass species, showed viscous behavior with dominant *G*″ only in the low ω, while the *G*″ deflected at elevated ω and an intersection of the *G*′ and *G*″ was observed. In the area where the *G*′ reached a higher value than *G*″, the solutions reacted as an elastic body. The observed viscoelastic properties indicated development of an intermicellar network with mechanically active cross-links. Moreover, the elastic response was stronger and the crossover between the *G*′/*G*″ occurred at lower ω for the networks with a higher density of micellar junctions and, therefore, longer relaxation time. As indicated by the steady shear behavior, the oscillatory shear study confirmed that the network of PRE_1.1_PRI(0.9) at a concentration of 20 wt % had the highest association degree among the IFHEURs based on PRE_1.1. Similarly, the elastic response observed for the solution of PRE_1.2_PRI(1.2) at 10 wt % was more pronounced compared to that for PRE_1.1_PRI(0.9) and occurred nearly over the whole ω range (Figure 5, left). Moreover, the *G*′ approached a constant value at elevated ω and the solution exhibited a short rubbery plateau, pointing toward dominating elastic behavior due to more extensive intermolecular associations. This effect was enhanced when the concentration of both IFHEURs based on the shorter PRE_1.2 prepolymer was increased to 20 wt %, as the *G*′ reached a higher value than *G*″ independently of the ω and the evident rubbery plateau was elongated. It can be assumed that under these conditions the strong associative cross-linking enabled formation of a complete physical network and yielded a solution with strong elastic behavior and resistance to deformation. On the contrary, the shorter chains of PRE_1.2_PRI(1.0) compared to those of PRE_1.2_PRI(1.2) were insufficient to build considerable intermicellar connections at a concentration of 10 wt %. The diluted solution displayed only viscous behavior, which corresponded to the low viscosity observed in the steady shear measurements.

These results indicated that the rheological behavior of the obtained IFHEURs was determined by their molar mass and the number of hydrophobes and their spacing between the hydrophilic segments. It was evident that the optimal degree of chain extension and denser packing of the hydrophobic tails in the core of the molecules promoted the formation of more developed physical networks.

## Conclusions

An innovative concept to prepare an eco-friendly alternative to hydrophobically modified ethoxylated urethane (HEUR) associative thickeners was proposed. The toxic reactants were replaced with green chemicals, and efficient, solvent-free reactive extrusion (REX) was applied to obtain sustainable isocyanate-free hydrophobically modified ethoxylated poly(hydroxy-urethane)s (IFHEURs). The green synthesis route started using CO_2_ as a precursor for the hydroxy-urethane bonds, fixing a CO_2_ molecule into each functional group of the five-membered poly(ethylene glycol) bis(cyclic carbonate) (BCC). The mild conditions required for aminolysis of BCC with 4,7,10-trioxa-1,13-tridecanediamine allowed the use of a standard batch reactor to produce the reactive hydrophilic segments—cyclic carbonate-terminated poly(hydroxy-urethane) PHU prepolymers. The following chain extension of the prepolymers with a biobased hydrophobic diamine PRIAMINE 1075, and thus the amphiphilic structure of the IFHEURs, was achieved efficiently and without using a solvent, thanks to the REX synthesis method.

The molar ratio between the reactants during each polymerization step was adjusted to obtain varied chain lengths of the PHU prepolymers and an optimal architecture of the final IFHEURs. Spectroscopic analysis confirmed that IFHEURs were obtained with a varied number of terminal and pendant hydrophobic groups built into a single chain. The gradual increase of the molar ratio between the PRI and PHU prepolymers led to a correspondingly higher conversion of the cyclic carbonate groups and a higher molar mass of the resulting IFHEURs with *M̅*_*w*_ in the range of 24,000–46,000 g·mol^–1^. At the stoichiometric amount of the reactants, the concentration of free cyclic carbonates was reduced to even 1.0 mol %, while the maximal conversion was reached within only 2 h of the REX process. Thus, using an extruder instead of a standard batch reactor benefited the efficiency of the reaction through enhanced mixing of the polymerizing melt and more homogeneous heat distribution.

The unique architecture of the obtained IFHEURs, containing both terminal and inner hydrophobic groups, had a crucial impact on their rheological performance in aqueous solutions. Both steady shear and oscillatory measurements confirmed that the IFHEUR molecules with sufficient chain length associated into mechanically active intermicellar cross-links. It is expected that the large species, containing multiple hydrophobic centers along the molecule, play a dominant role in the thickening mechanism and form superbridges extended across the network. Furthermore, a change in the spacing between the hydrophobes, determined by the length of the used hydrophilic PHU prepolymer, modulated the strength of the network and allowed it to cover a broad range of viscoelastic behavior.

The demonstrated approach thus allows a shift from environmentally burdening isocyanates to amine chain extenders or end-cappers, while the variety of available biobased amines opens perspectives for a new architecture and material performance. Moreover, the presence of pendant hydroxyl groups in each urethane unit of IFHEURs can facilitate their solubility in water and provide an option for further structural modifications, bringing unique properties not attainable by the conventional HEURs. With growing access to commercial cyclic carbonate monomers, the isocyanate-free synthesis pathway toward IFHEURs offers tremendous potential to deliver sustainable rheological modifiers for waterborne systems.

## Supporting Information Available

Additional experimental details, including ^1^H and ^13^C NMR spectra, FT-IR spectra, MALDI-ToF mass spectra, and viscosity measurements (PDF)
