# Supplementary material for: Reactive Extrusion Synthesis of Biobased Isocyanate-Free Hydrophobically Modified Ethoxylated Urethanes with Pendant Hydrophobic Groups
Source: ACS Sustain Chem Eng. 2022 Aug 22;10(35):11627–40. doi: 10.1021/acssuschemeng.2c03535 (PMC9450225; doi:10.1021/acssuschemeng.2c03535)
Supplement: Supplementary file 1 — sc2c03535_si_001.pdf [file sc2c03535_si_001.pdf]

Supporting Information

for

Reactive extrusion synthesis of bio-based isocyanate-free hydrophobically modified ethoxylated urethanes with pendant hydrophobic groups

Dominik Wołosz[a,*], Aleksandra Marta Fage[b], Paweł Grzegorz Parzuchowski[a], Aleksandra Świderska[a], and Robert Brüll[c]

[a] – *Warsaw University of Technology, Faculty of Chemistry, Noakowskiego 3, 00-664 Warsaw, Poland*

[b] – *Fraunhofer Institute for Chemical Technology ICT, Joseph-von-Fraunhofer-Straße 7, 76327 Pfinztal, Germany*

[c] – *Fraunhofer Institute for Structural Durability and System Reliability LBF, Bartningstraße 47, 64289 Darmstadt, Germany*

* Corresponding author: dominik.wolosz.dokt@pw.edu.pl

**Number of Pages**	**23**
**Number of Figures**	**31**
**Number of Tables**	**3**

Table of Contents:

1. Synthesis of poly(ethylene glycol) bis(cyclic carbonate) (BCC). S3

2. Synthesis of hydrophilic cyclic carbonate-terminated poly(hydroxy-urethane) prepolymers. S5

3. Synthesis of isocyanate-free hydrophobically modified ethoxylated poly(hydroxy-urethane)s (IFHEURs) via the reactive extrusion process. S10

4. Calculations concerning the prepolymers and IFHEURs. S20

5. Viscosity measurements in the online capillary viscometer of the extruder during REX synthesis of IFHEURs. S22

1. Synthesis of poly(ethylene glycol) bis(cyclic carbonate) (BCC).

1.1. FT-IR

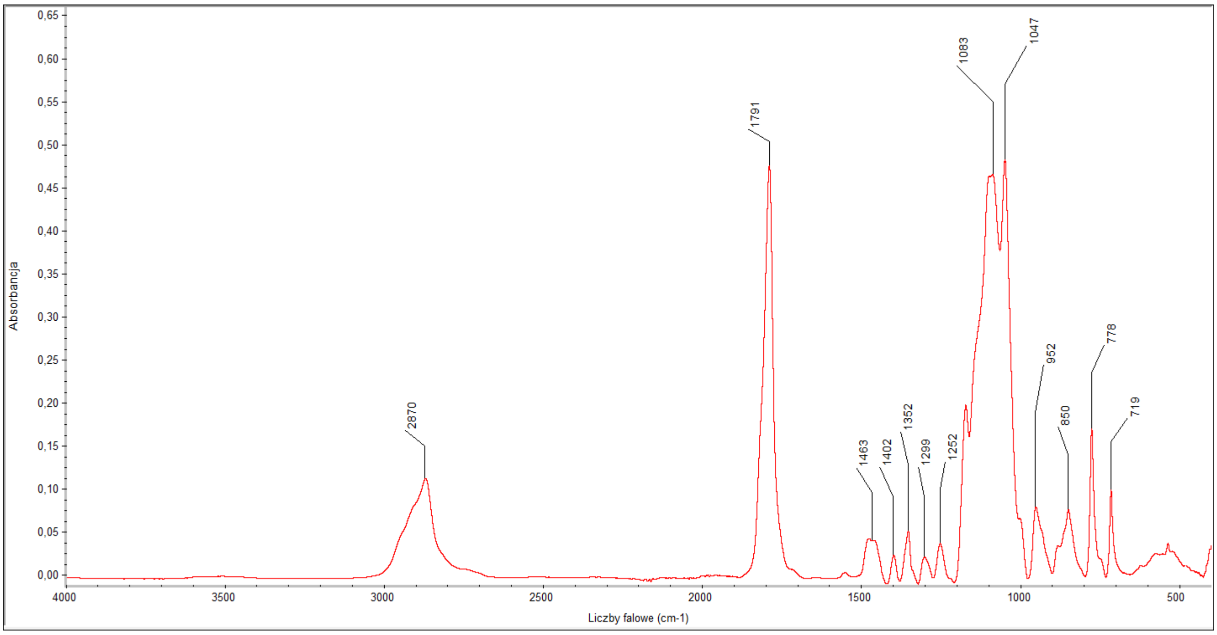

Fig. S1. FT-IR spectrum of **BCC**.

1.2. ^{1}H NMR

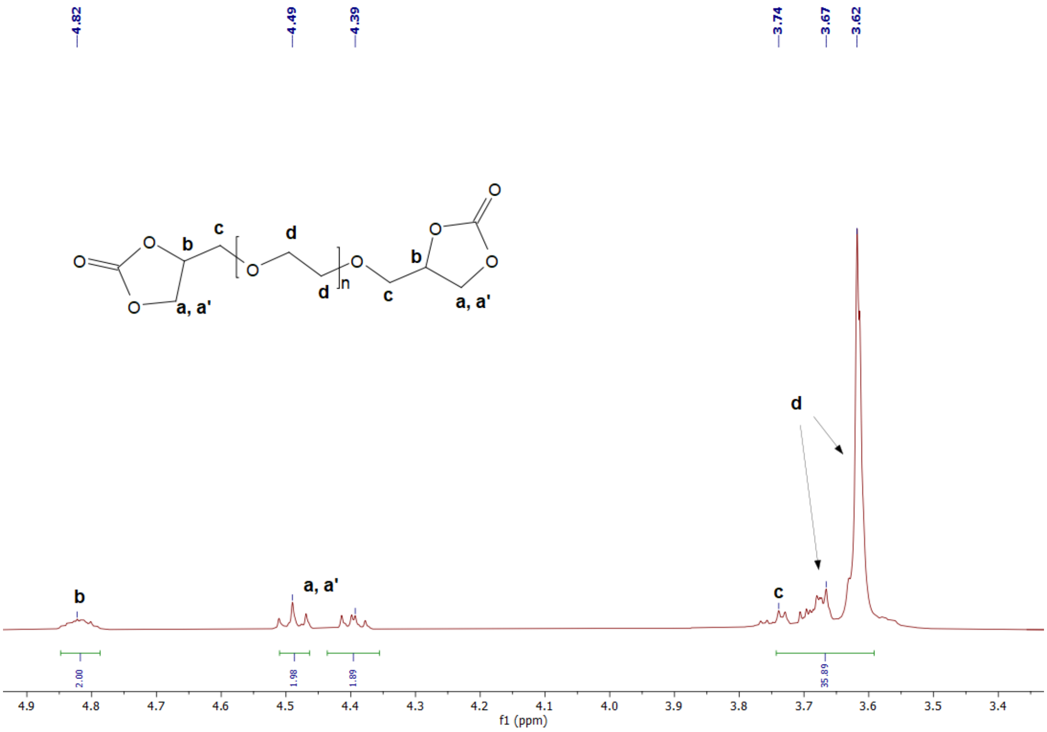

Fig. S2. ^{1}H NMR spectrum of **BCC**.

1.3. ^{13}C NMR

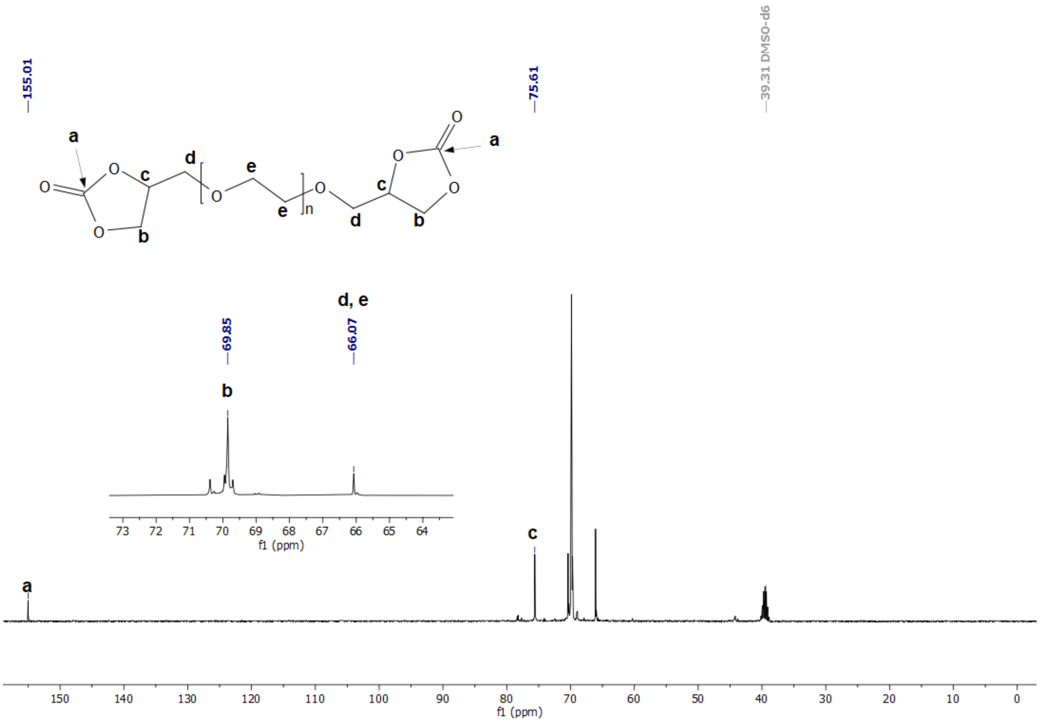

Fig. S3. ^{13}C NMR spectrum of **BCC**.

2. Synthesis of hydrophilic cyclic carbonate-terminated poly(hydroxy-urethane) prepolymers.

2.1. Formulations

Table S1. The amounts of reagents used during the synthesis of the **prepolymers**.

**Prepolymer**	**BCC**[a]		**TTDDA**		**BCC : TTDDA molar ratio /-**	$\bar{M}_{n(theo)}$[b] **/g·mol^{-1}**
	**m /g**	**n /mol**	**m /g**	**n /mol**		
**PRE_1.1**	86.350	0.151	30.322	0.138	1.1 : 1.0	8 500
**PRE_1.2**	70.670	0.124	22.748	0.103	1.2 : 1.0	4 500

[a] – calculated based on Fig. S2 and Eq. S1.
[b] – calculated based on Eq. S2.

2.2. FT-IR

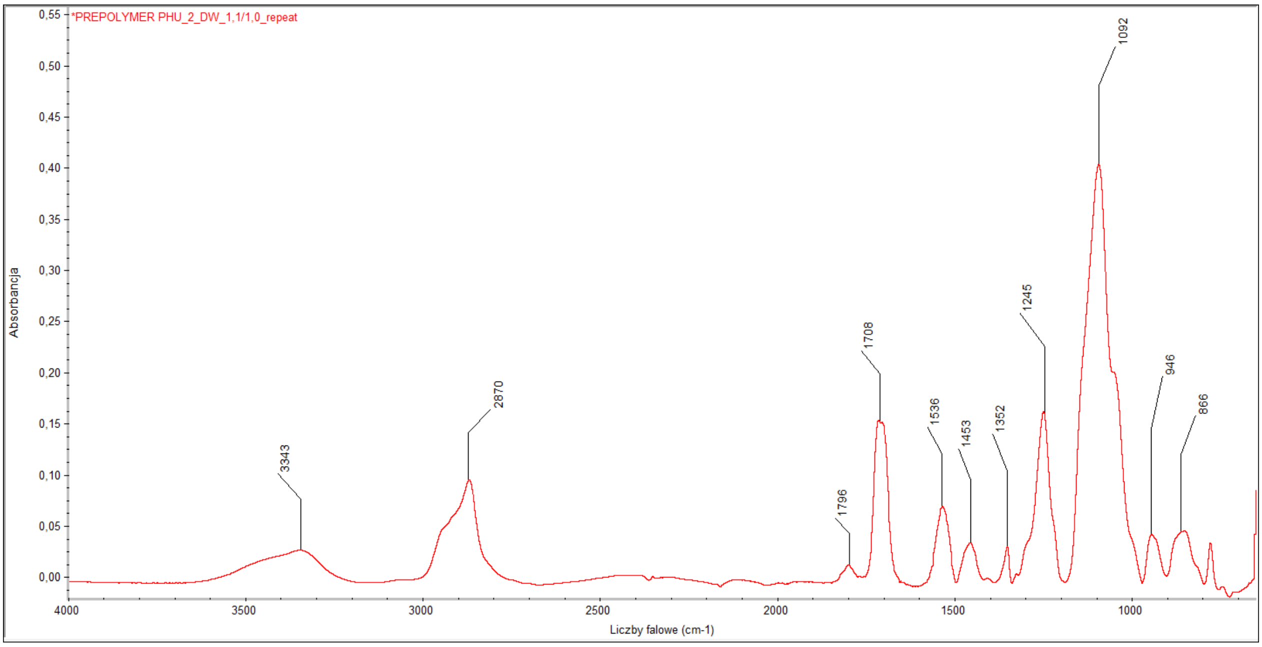

Fig. S4. FT-IR spectrum of the **PRE_1.1**.

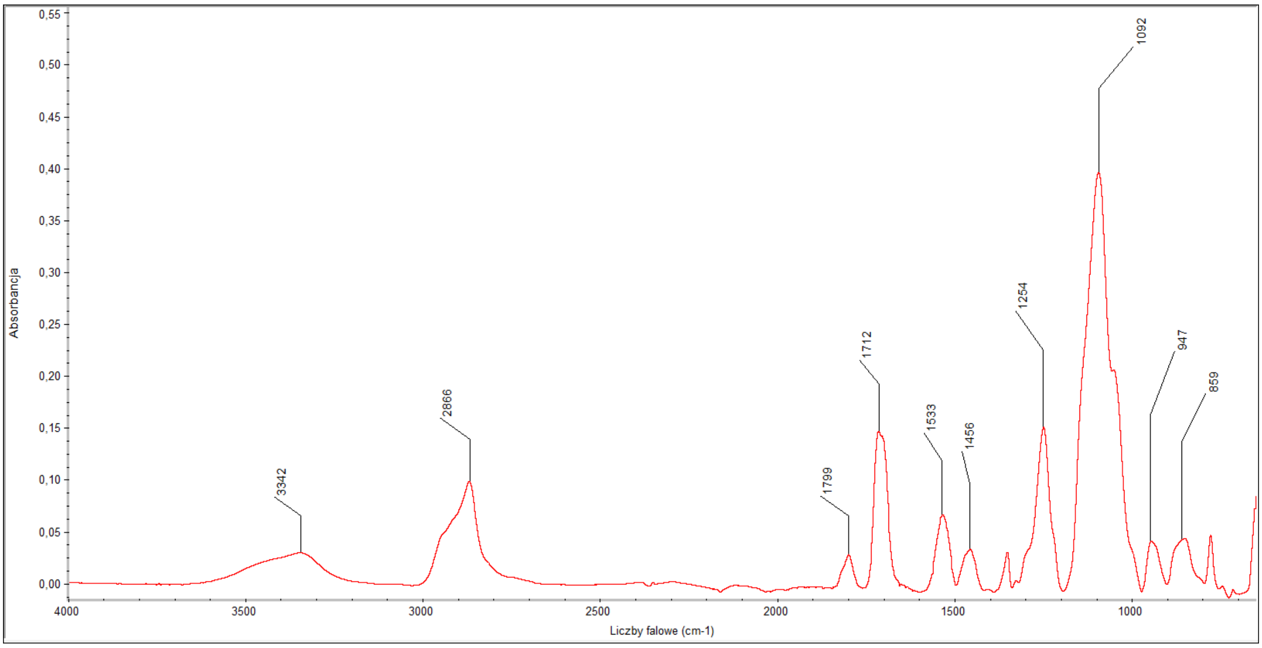

Fig. S5. FT-IR spectrum of the **PRE_1.2**.

2.3. ^{1}H NMR

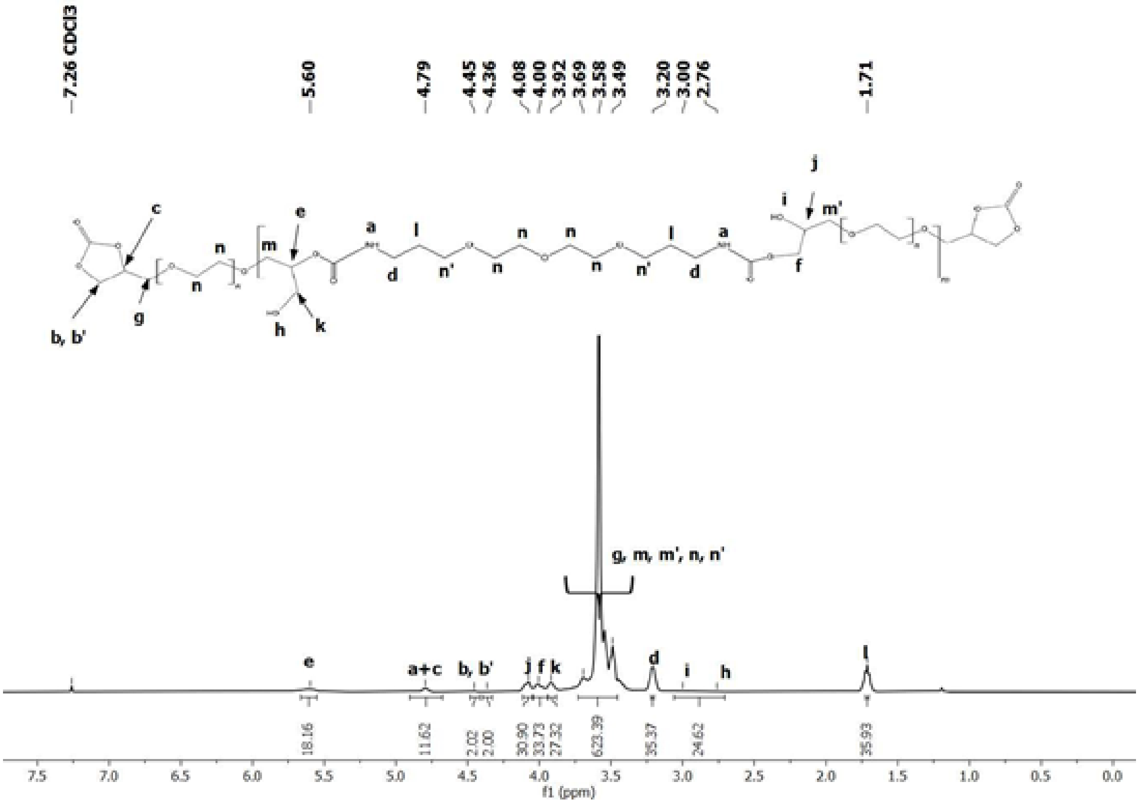

Fig. S6. ^{1}H NMR spectrum of the **PRE_1.1**.

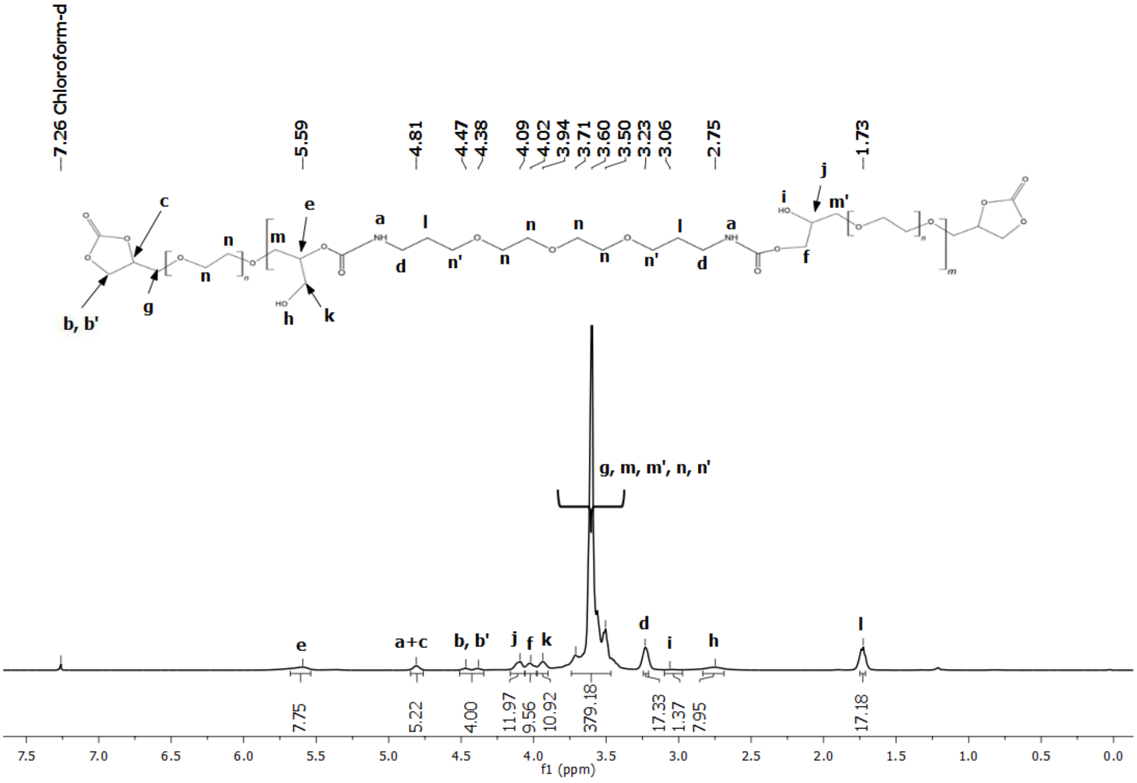

Fig. S7. ^{1}H NMR spectrum of the **PRE_1.2**.

2.4. ^{13}C NMR

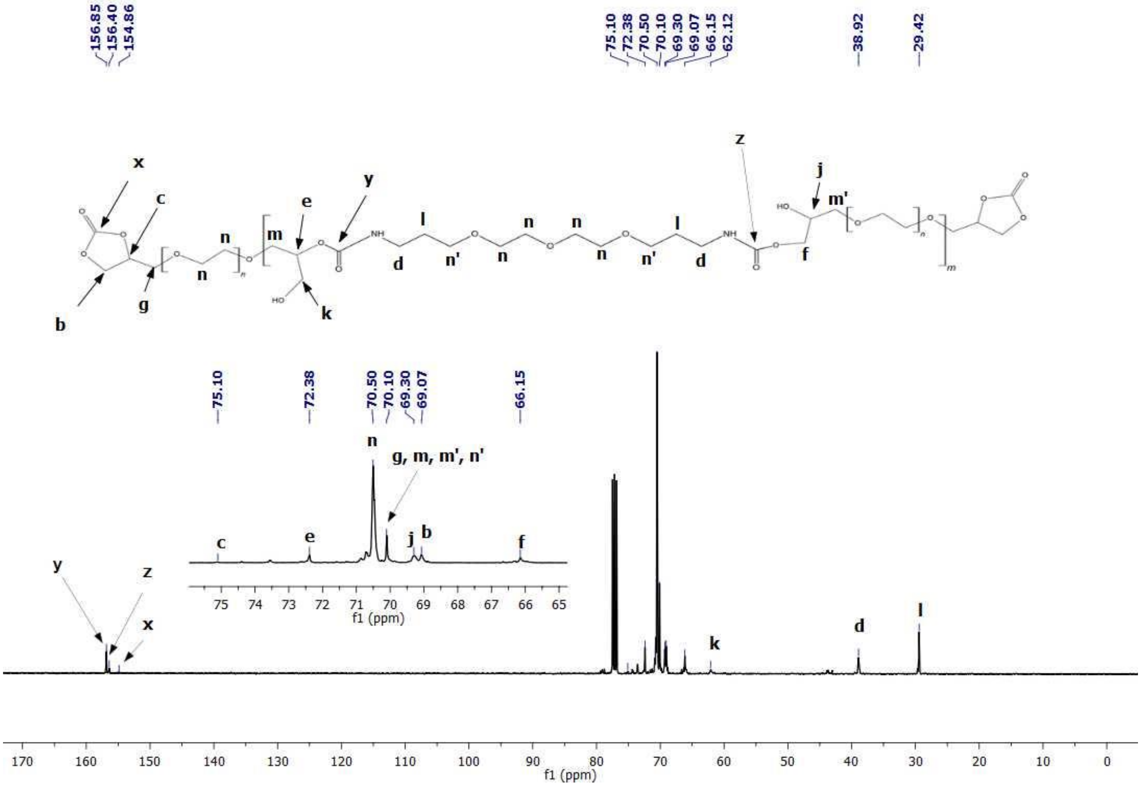

Fig. S8. ^{13}C NMR spectrum of the **PRE_1.1**.

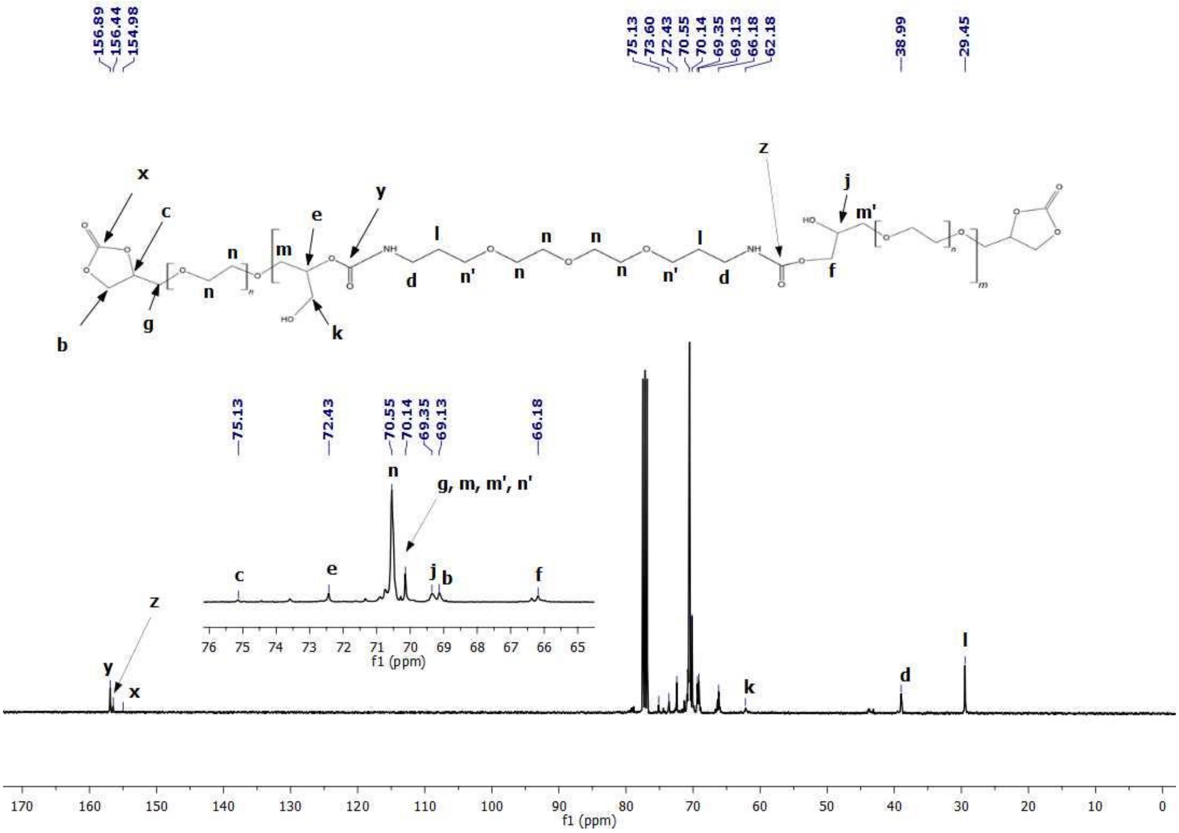

Fig. S9. ^{13}C NMR spectrum of the **PRE_1.2**.

2.5. MALDI-ToF mass spectrometry

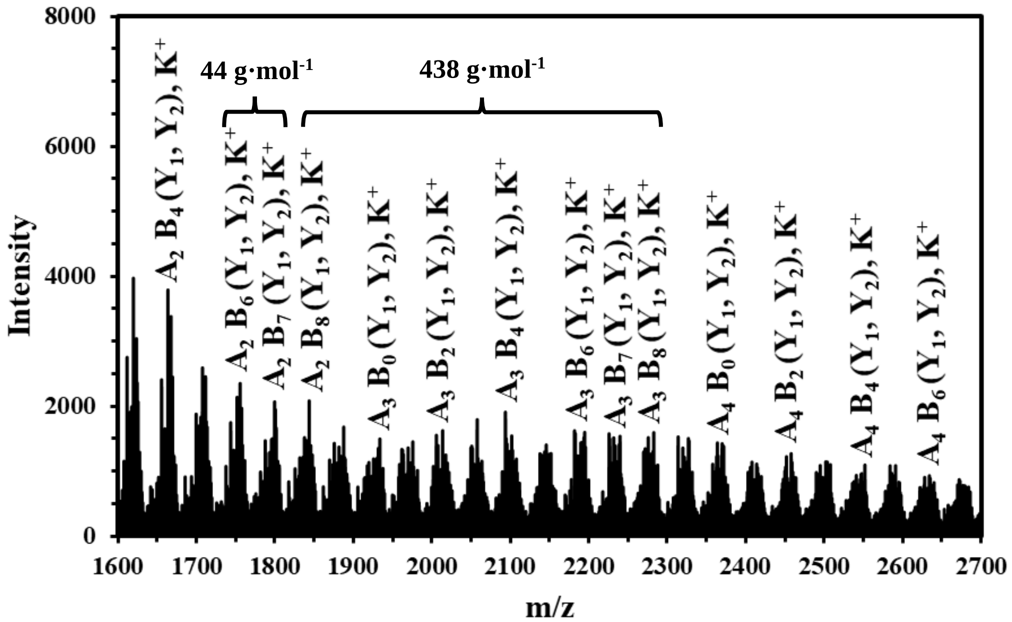

Fig. S10. MALDI-ToF mass spectrum of **PRE_1.1**.

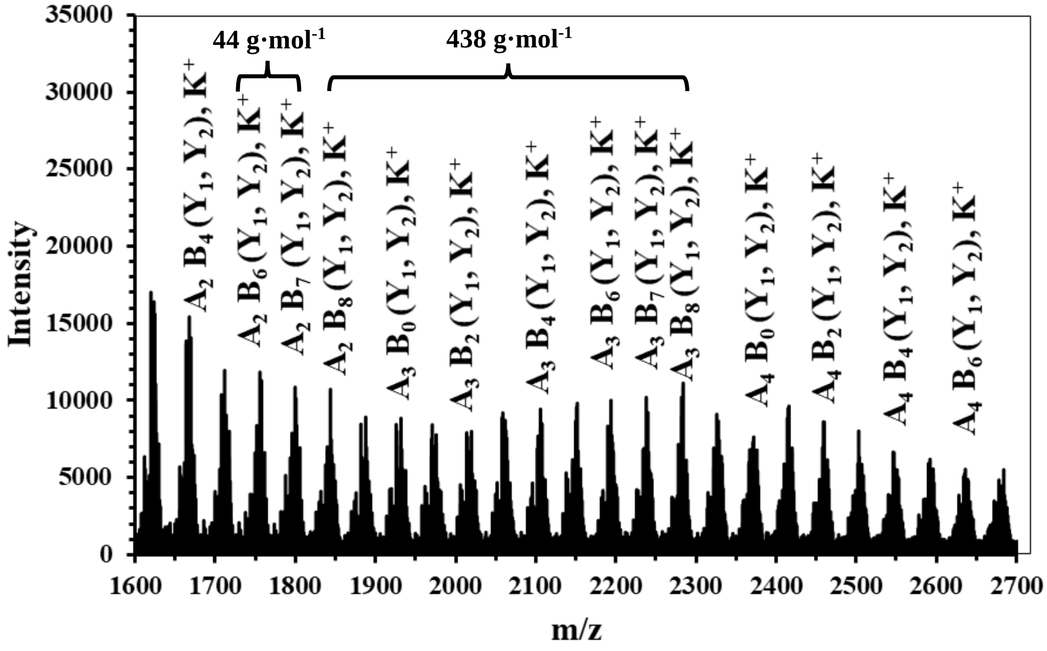

Fig. S11. MALDI-ToF mass spectrum of **PRE_1.2**.

3. Synthesis of isocyanate-free hydrophobically modified ethoxylated poly(hydroxy-urethane)s (IFHEURs) via the reactive extrusion process.

3.1. Formulations

Table S2. The details concerning the synthesis formulation of the **IFHEURs** and the reaction conditions.

Sample	Prepolymer type	Prepolymer[a]		PRI		PRE : PRI molar ratio /-	Reaction temp. /°C
		m /g	PRE /mmol	m /g	n /mmol		
**PRE_1.1_PRI(0.8)**	PRE_1.1	7.3500	0.85	0.3515	0.64	1.0 : 0.8	120
**PRE_1.1_PRI(0.9)**	PRE_1.1	7.3000	0.87	0.4125	0.75	1.0 : 0.9	120
**PRE_1.1_PRI(1.0)**	PRE_1.1	7.4000	0.88	0.4825	0.88	1.0 : 1.0	120
**PRE_1.1_PRI(1.0) _100C**	PRE_1.1	7.3500	0.85	0.4793	0.86	1.0 : 1.0	100
**PRE_1.2_PRI(1.0)**	PRE_1.2	7.3200	1.63	0.9487	1.70	1.0 : 1.0	120
**PRE_1.2_PRI(1.2)**	PRE_1.2	7.3500	1.63	1.0992	1.98	1.0 : 1.2	120

[a] – molar mass calculated based on Eq. S2

3.2. FT-IR

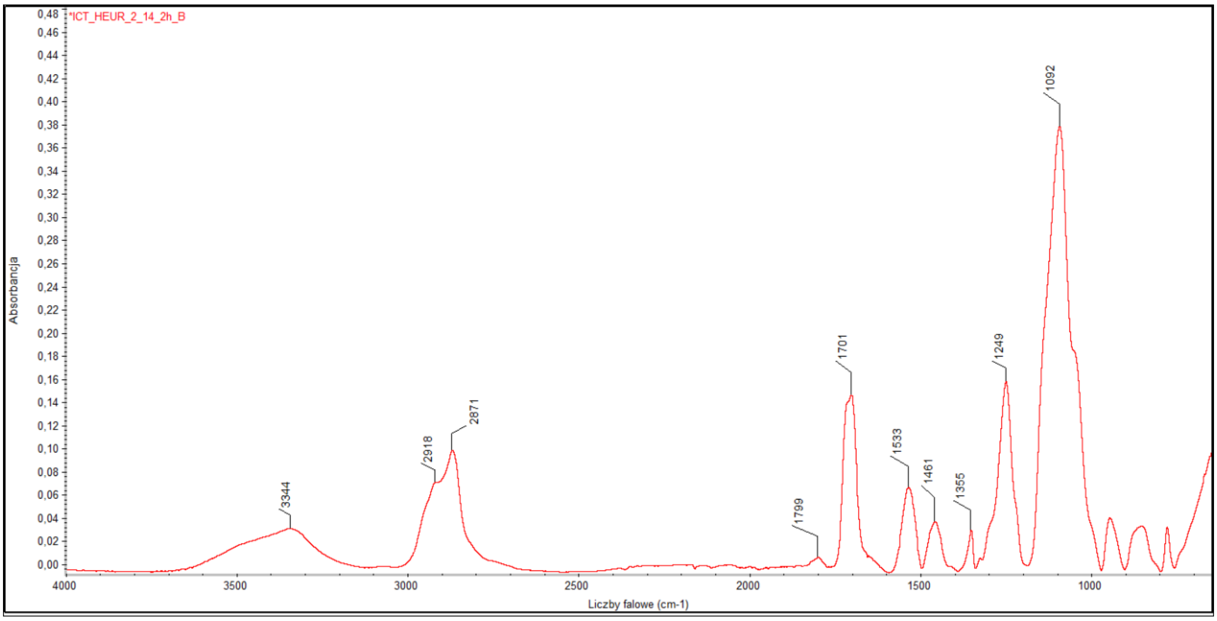

Fig. S12. FT-IR spectrum of the **PRE_1.1_PRI(0.8)**.

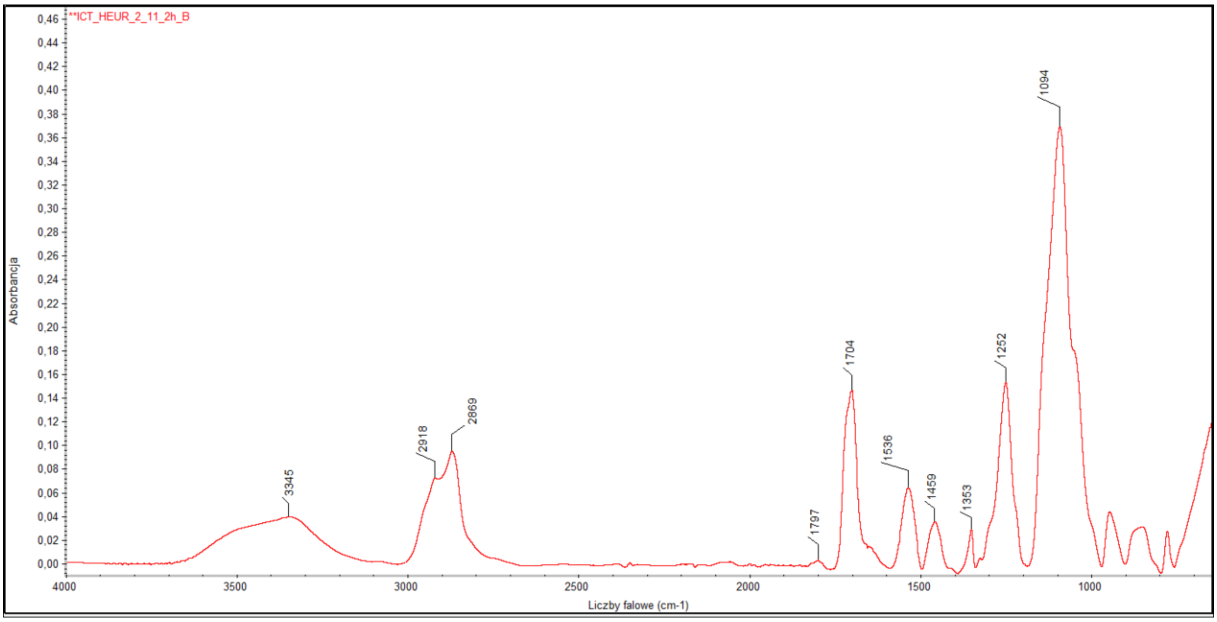

Fig. S13. FT-IR spectrum of the **PRE_1.1_PRI(0.9)**.

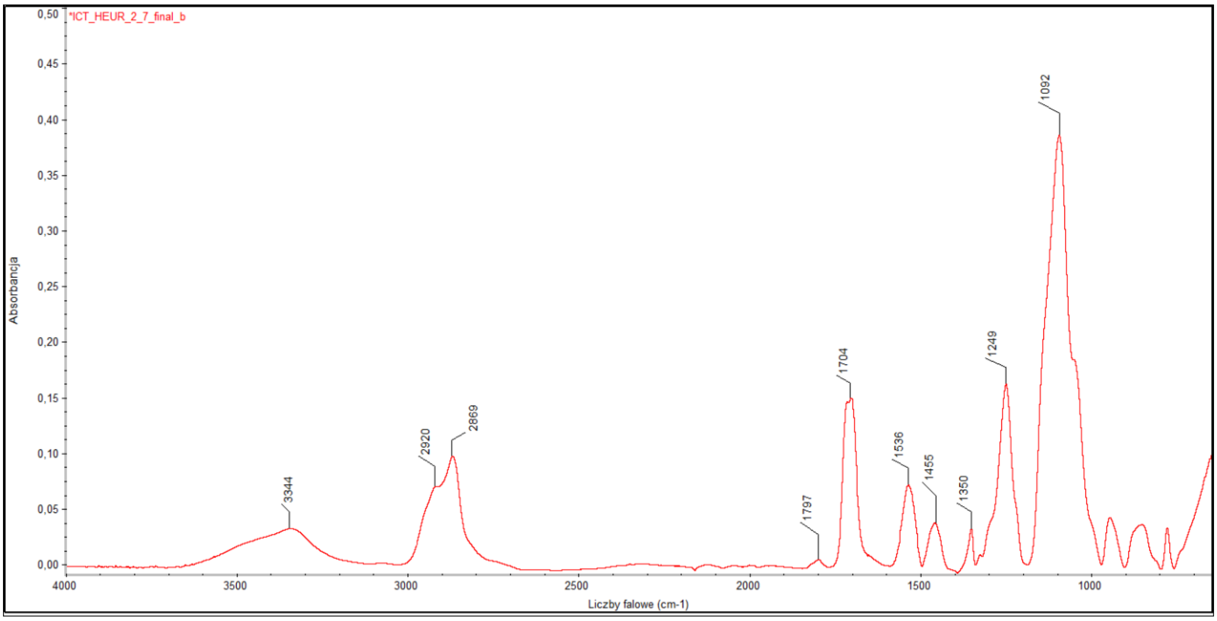

Fig. S14. FT-IR spectrum of the **PRE_1.1_PRI(1.0)**.

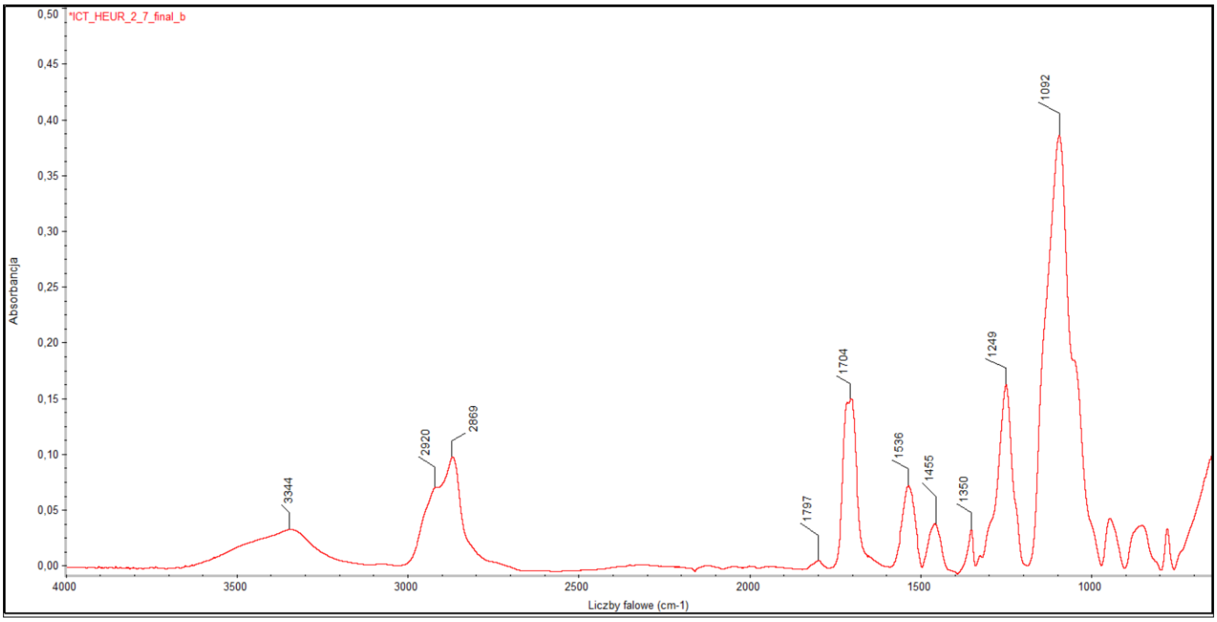

Fig. S15. FT-IR spectrum of the **PRE_1.1_PRI(1.0)_100C**.

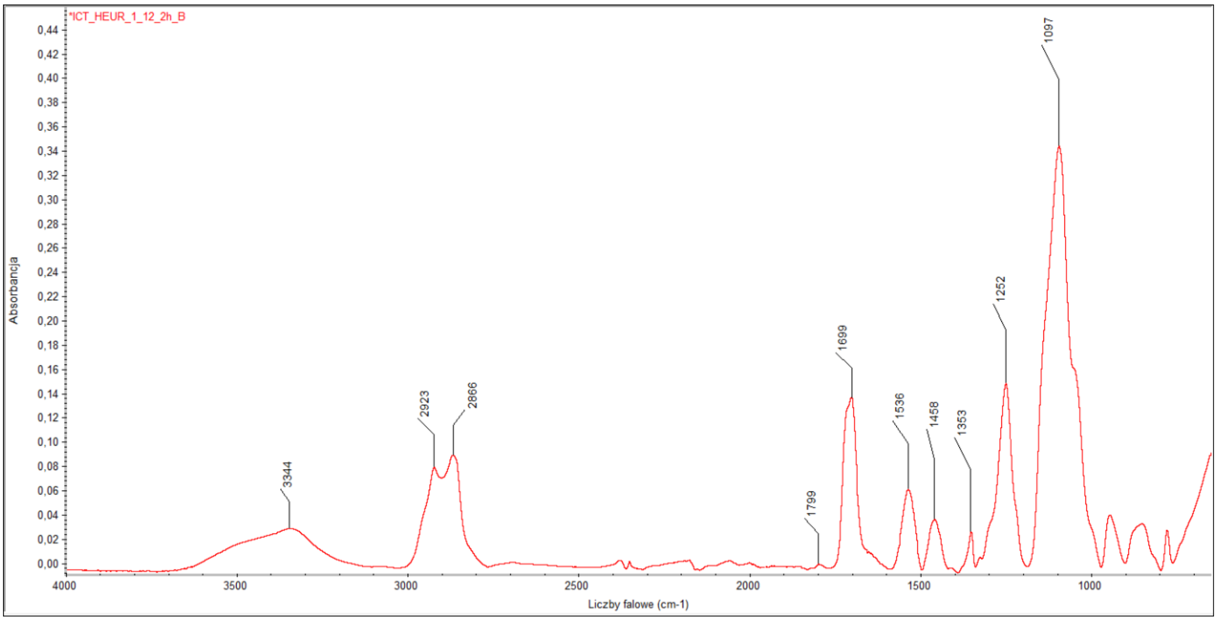

Fig. S16. FT-IR spectrum of the **PRE_1.2_PRI(1.0)**.

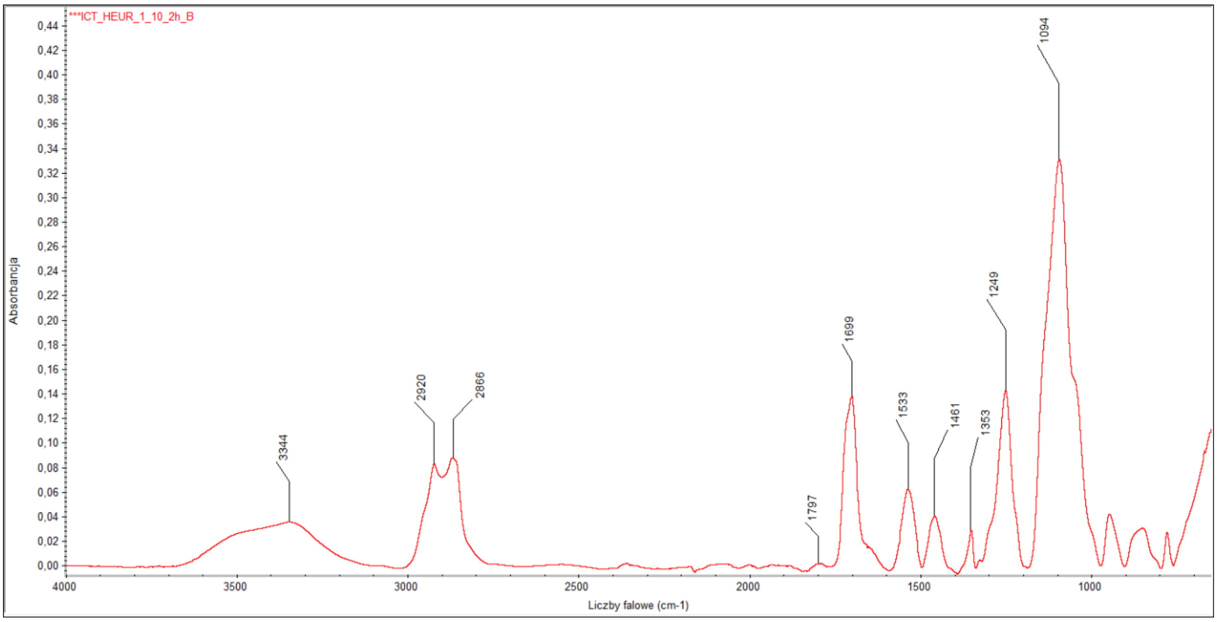

Fig. S17. FT-IR spectrum of the **PRE_1.2_PRI(1.2)**.

3.3.¹H NMR

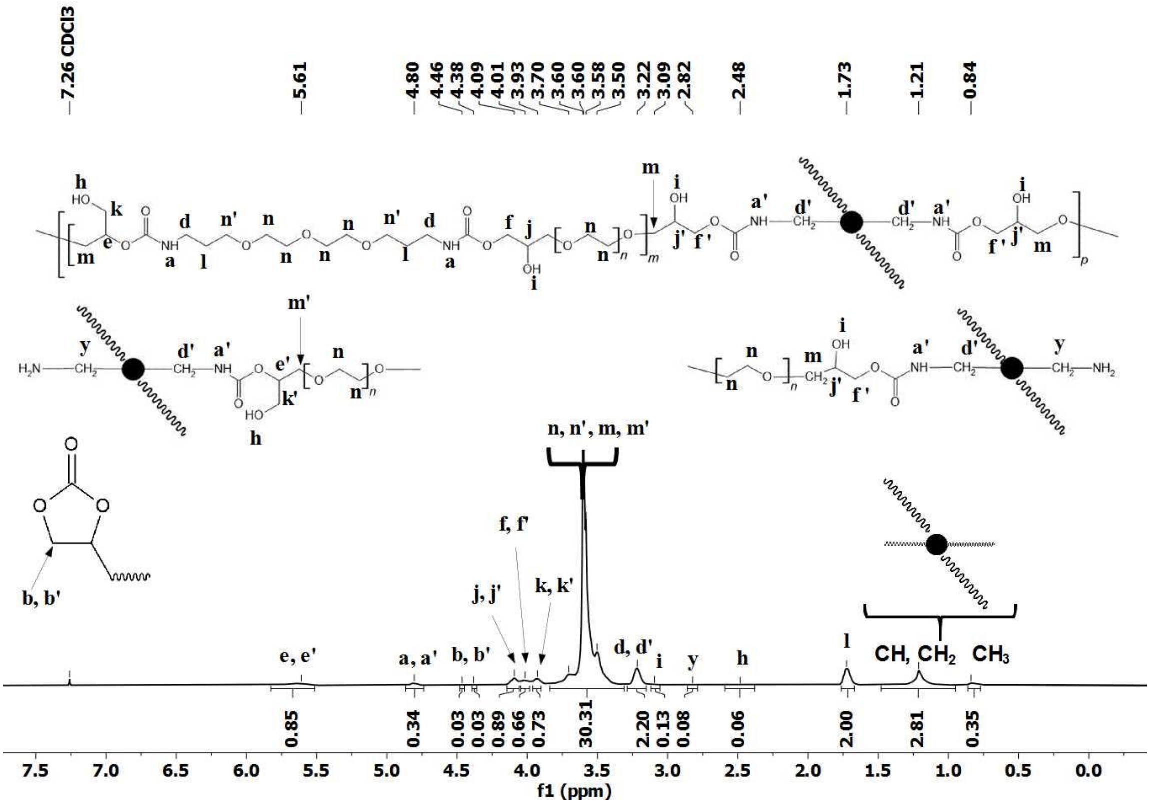

Fig. S18. ¹H NMR spectrum of the **PRE_1.1_PRI(0.8)**.

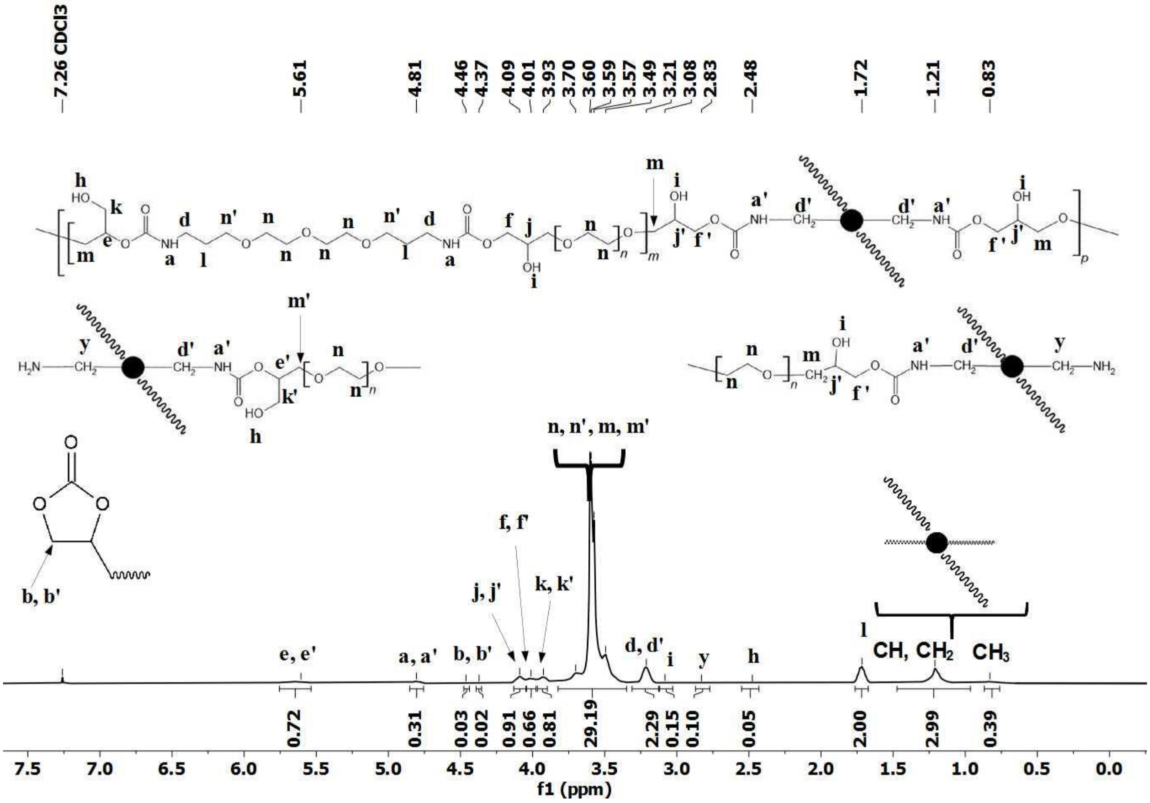

Fig. S19. ^{1}H NMR spectrum of the **PRE_1.1_PRI(0.9)**.

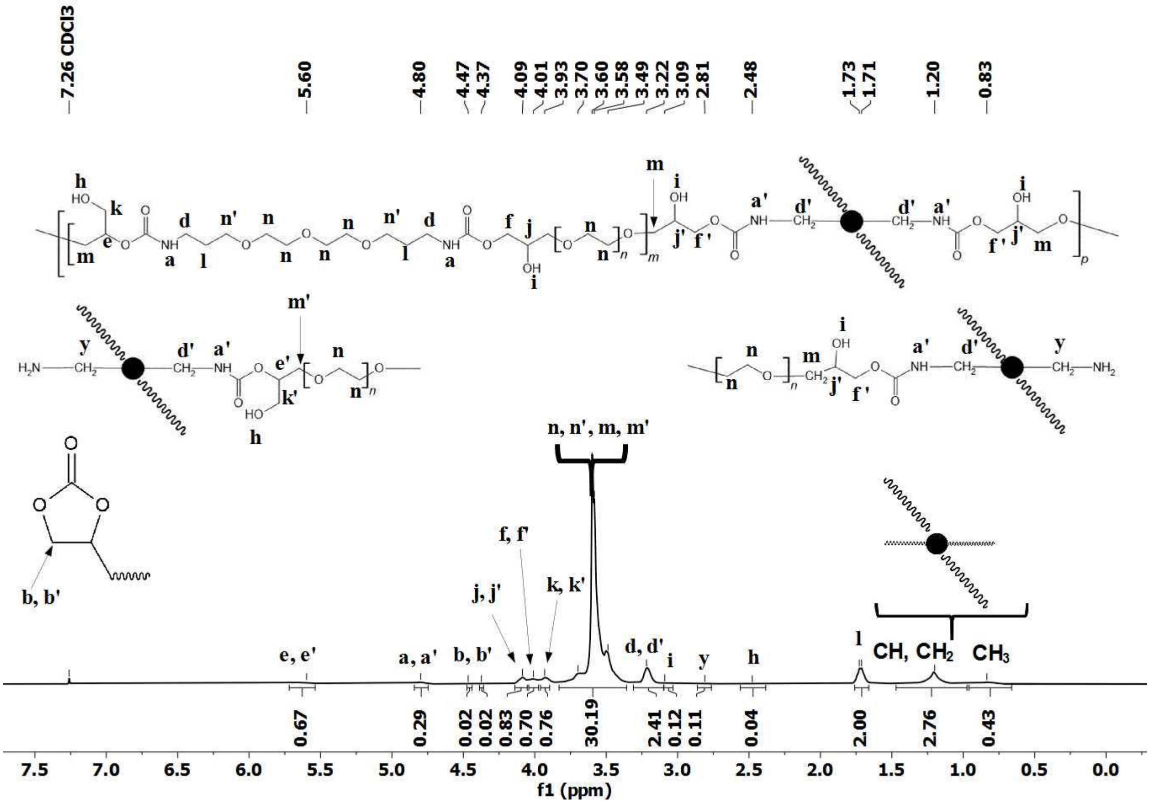

Fig. S20. ^{1}H NMR spectrum of the **PRE_1.1_PRI(1.0)**.

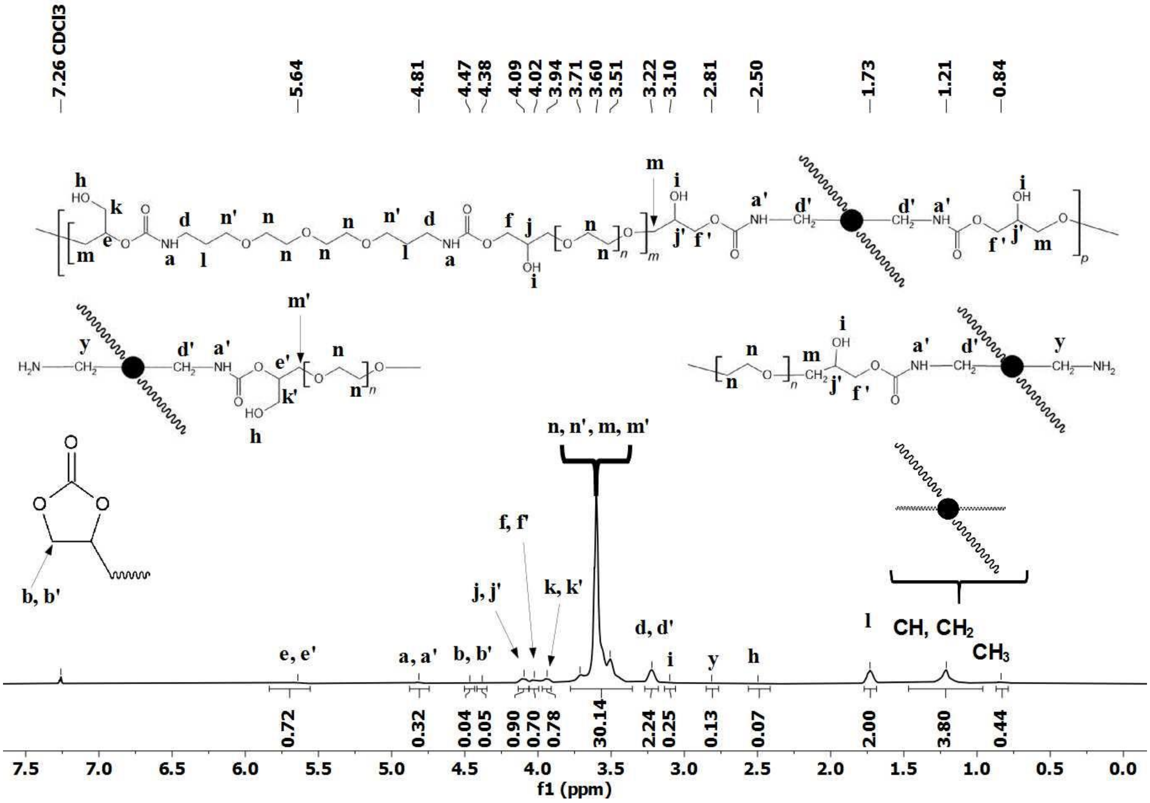

Fig. S21. ^{1}H NMR spectrum of the **PRE_1.1_PRI(1.0)_100C**.

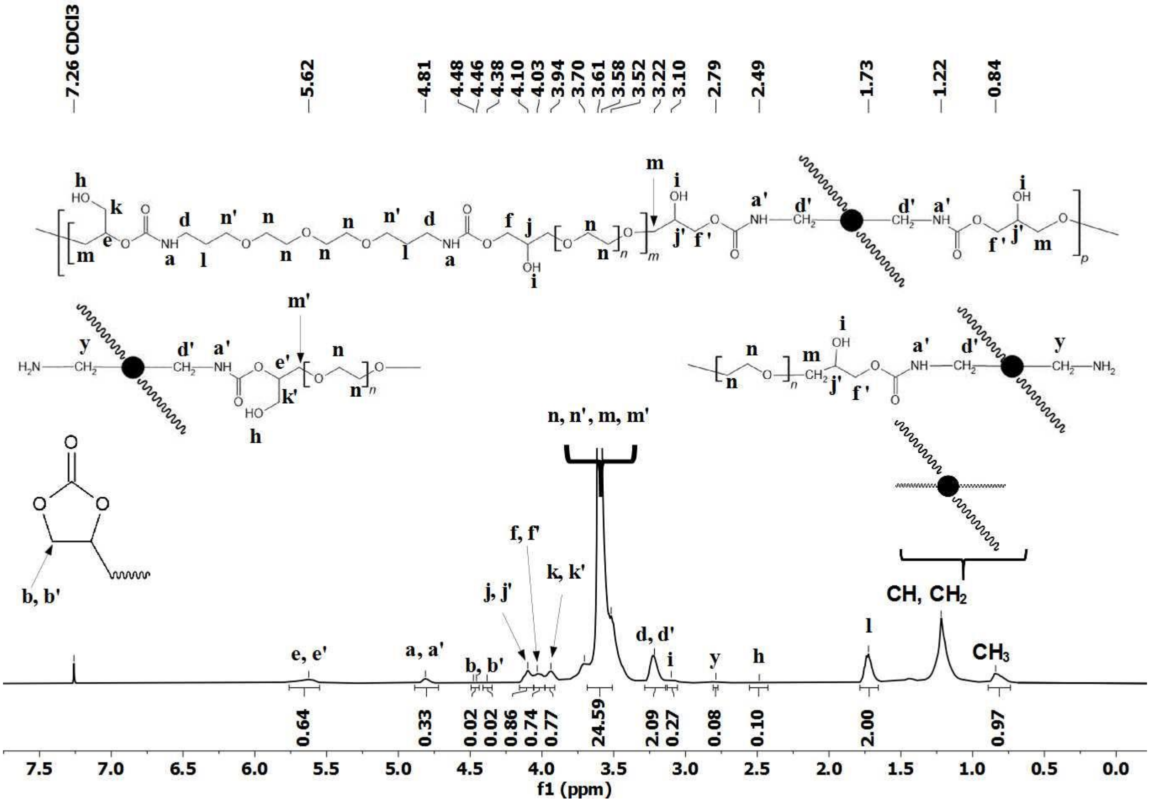

Fig. S22. ^{1}H NMR spectrum of the **PRE_1.2_PRI(1.0)**.

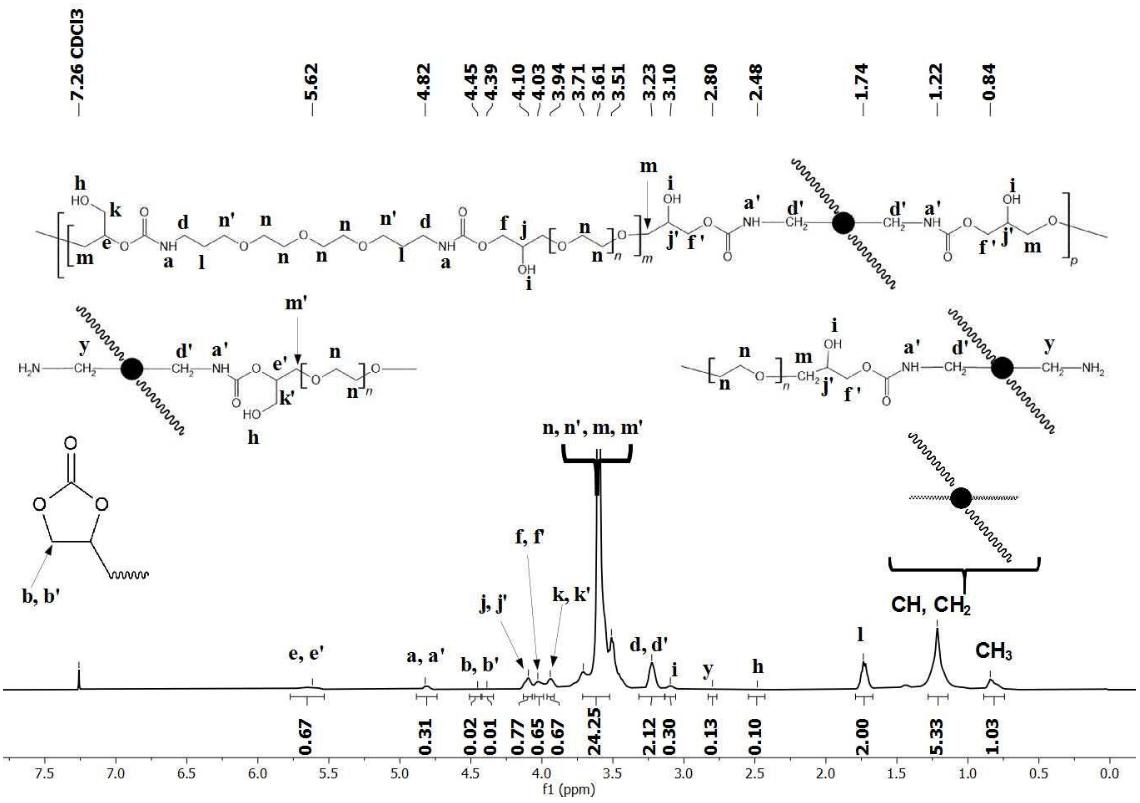

Fig. S23. ^{1}H NMR spectrum of the **PRE_1.2_PRI(1.2)**.

3.4. ^{13}C NMR

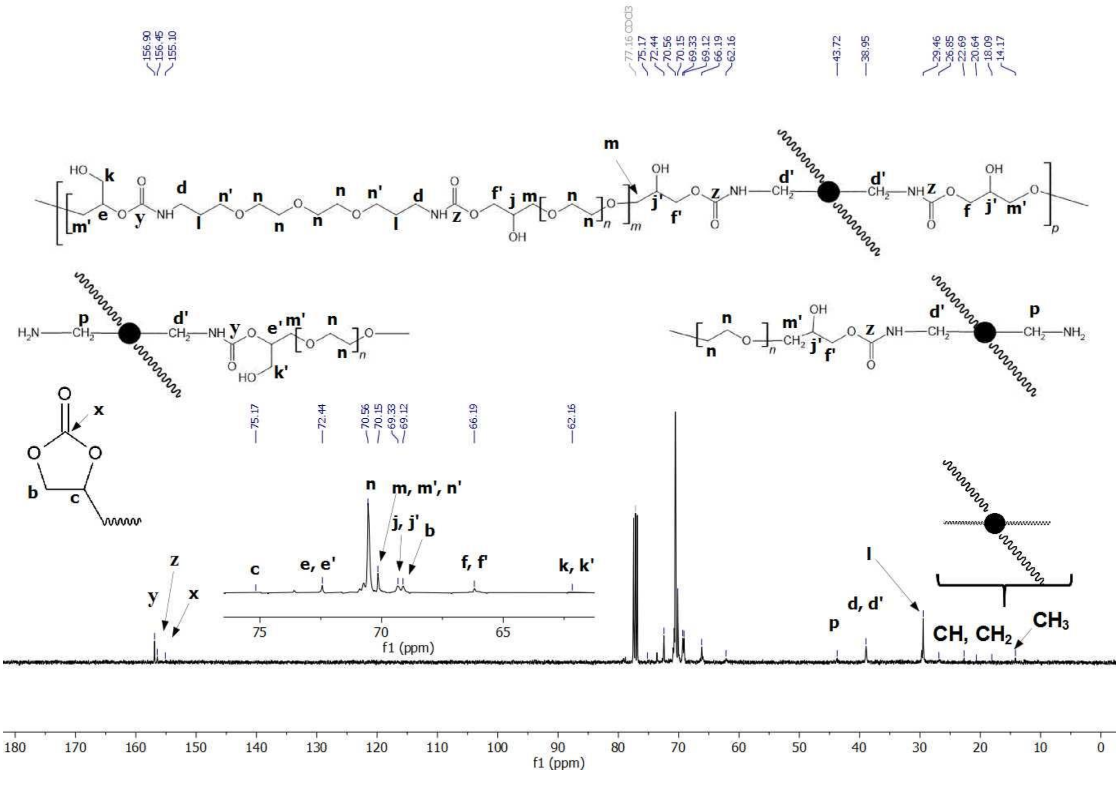

Fig. S24. ^{13}C NMR spectrum of the **PRE_1.1_PRI(0.8)**.

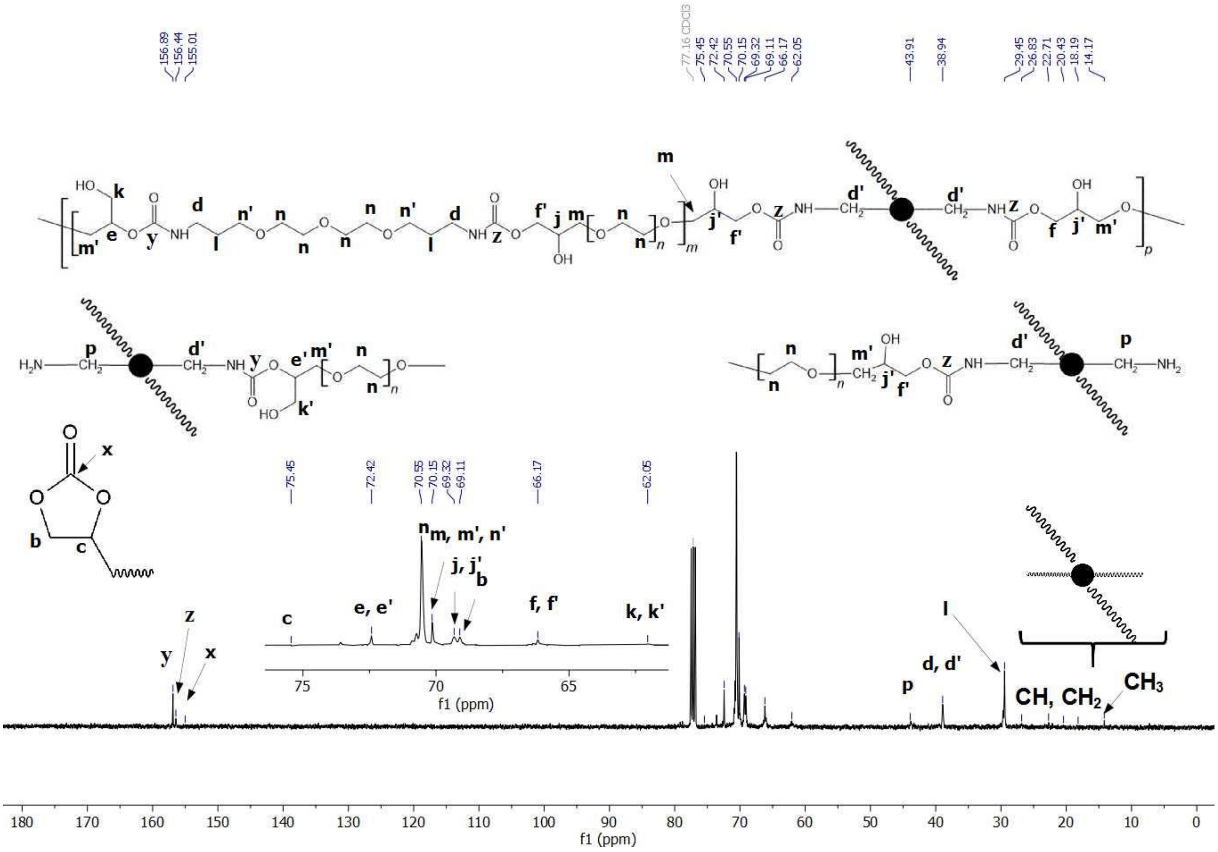

Fig. S25. ^{13}C NMR spectrum of the **PRE_1.1_PRI(0.9)**.

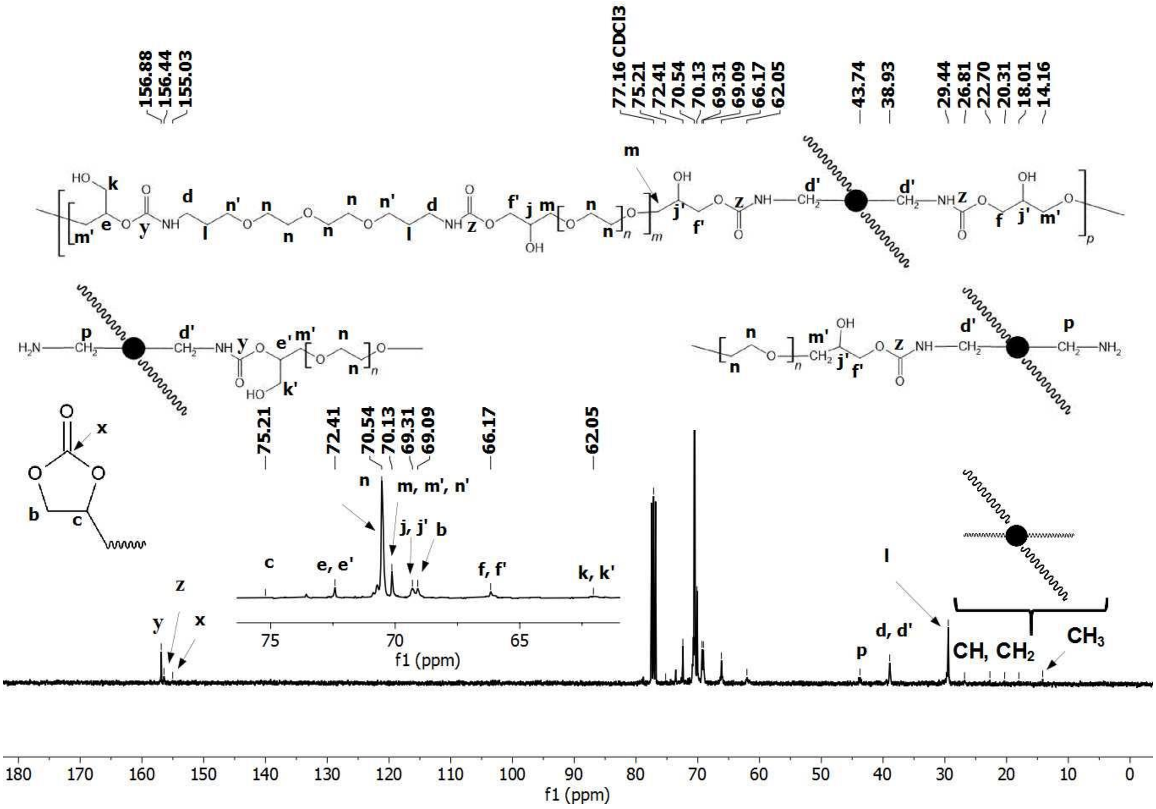

Fig. S26. ^{13}C NMR spectrum of the **PRE_1.1_PRI(1.0)**.

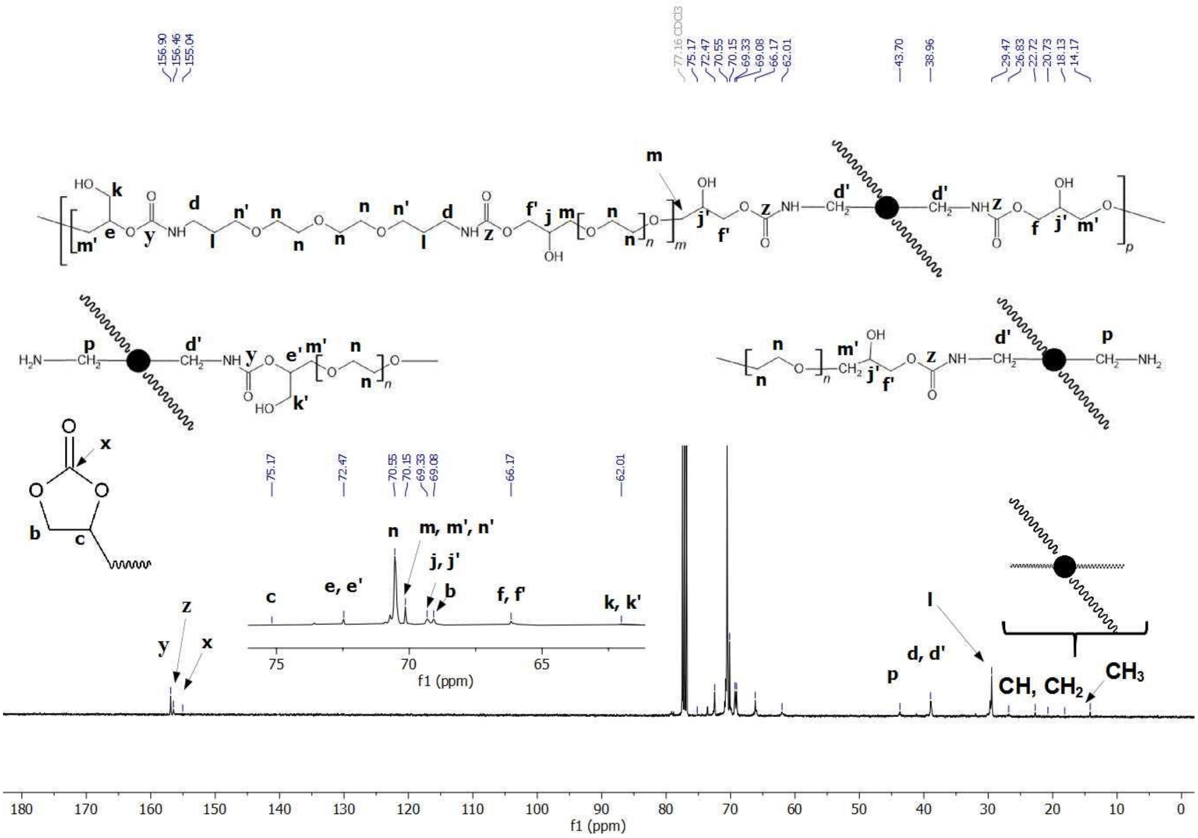

Fig. S27. ^{13}C NMR spectrum of the **PRE_1.1_PRI(1.0)_100C**.

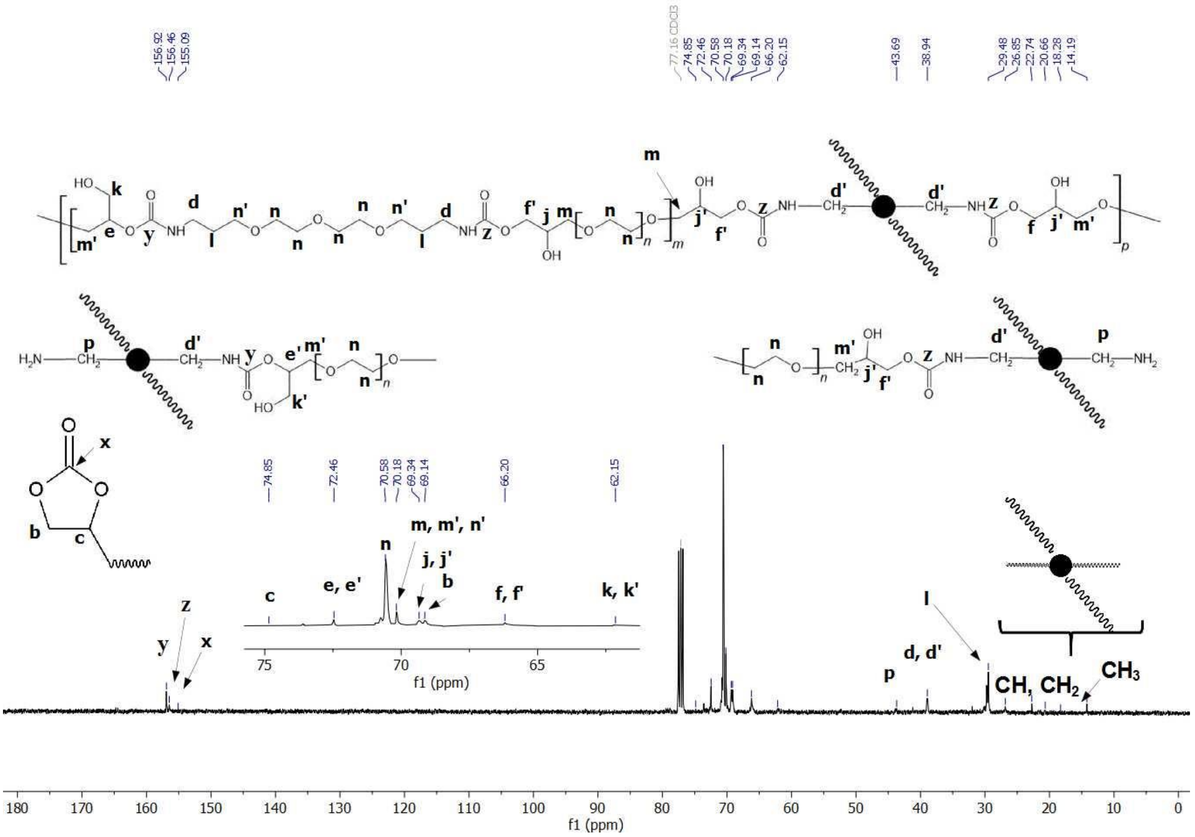

Fig. S28. ^{13}C NMR spectrum of the **PRE_1.2_PRI(1.0).**

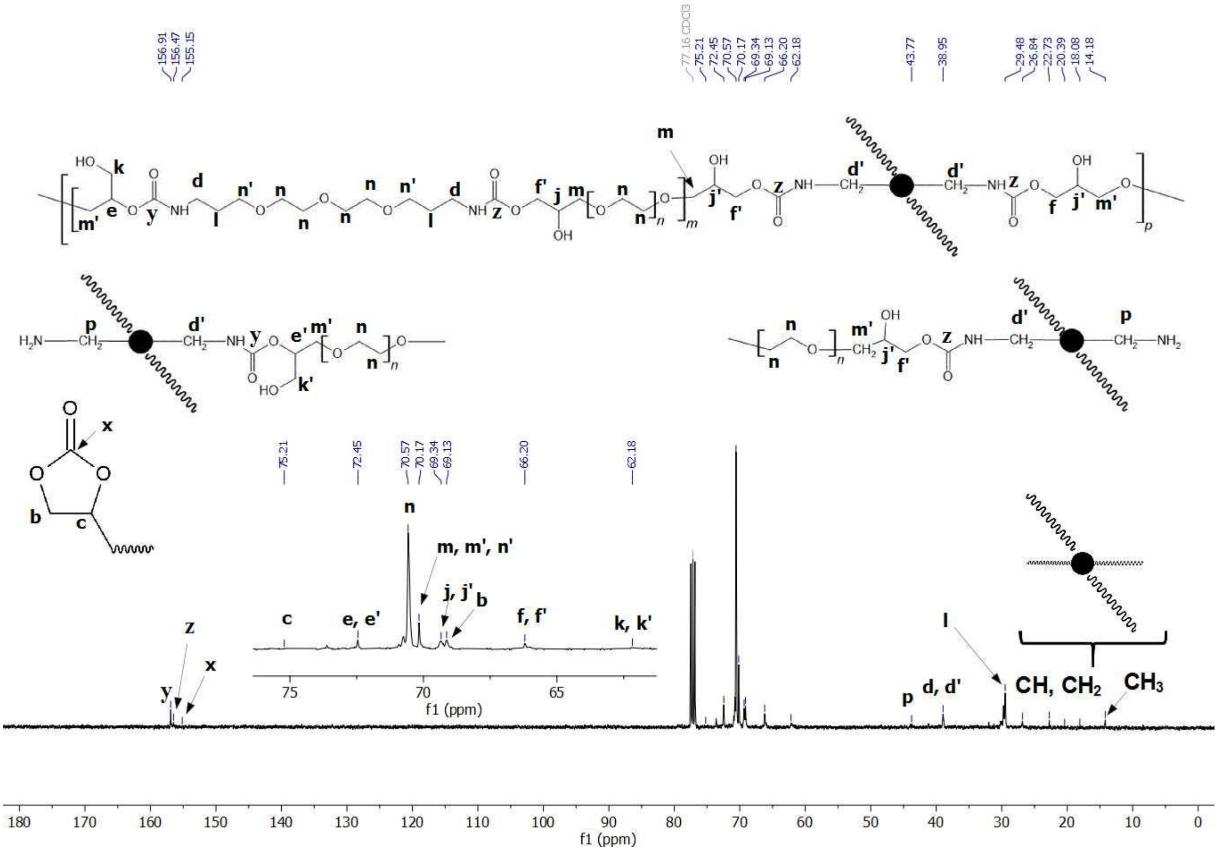

Fig. S29. ^{13}C NMR spectrum of the **PRE_1.2_PRI(1.2).**

4. Calculations concerning the prepolymers and IFHEURs.

The number-average molar mass ($\bar{M}_{n(NMR)}$) of **BCC** was calculated based on ^{1}H NMR spectra (**Fig. S2**) according to **Eq. S1**.

Eq. S1 $$\bar{M}_{n(NMR)\,[g\cdot mol^{-1}]} = \frac{\int c,d - 2\int b}{4} \cdot M_{mer} + M_{end\ group\ 1} + M_{end\ group\ 2}$$

The **M_{mer}**, **$M_{end\ group\ 1}$**, and **$M_{end\ group\ 2}$** equalled 44 g·mol^{-1}, 101 g·mol^{-1}, and 117 g·mol^{-1}, respectively.

The theoretical molar mass of **prepolymers** was calculated based on stoichiometry of the reaction between the **BCC** and **TTDDA** (Eq. S2).

Eq. S2 $$M_{theoret\,[g\cdot mol^{-1}]} = m \cdot M_{BCC} + n \cdot M_{TTDDA}$$

The **m** and **n** values equalled **11** and **10** for **PRE_1.1**, or **6** and **5** for **PRE_1.2**, which correspond to molar ratio of the BCC and TTDDA used in the synthesis. The **M_{BCC}** and **M_{TTDDA}** were **570** g·mol^{-1} and **220** g·mol^{-1}.

The number-average molar mass ($\bar{M}_{n(NMR)}$) of **prepolymers** was calculated based on ^{1}H NMR spectra (e.g. **Fig. S6**) according to **Eq. S3** and **Eq. S4**. The signal **b,b'** was used as a reference (integral intensity equals 4.00).

Eq. S3 $$m_{[-]} = \frac{\int_l}{n_l}$$

Eq. S4 $$\bar{M}_{n(NMR)\,[g\cdot mol^{-1}]} = m \cdot M_{mer} + M_{end\ group\ 1} + M_{end\ group\ 2}$$

The integral **∫l** corresponds to the intensity of the **l** signal (1.73 ppm at **Fig. S6**) coming from **CH_2**CH_2NHC(O)O groups. The number of protons in **l** group related to **n_l** symbols and equalled 4.00. The M_{mer}, $M_{end\ group\ 1}$, and $M_{end\ group\ 2}$ were equalled 785 g·mol^{-1}, 101 g·mol^{-1} and 469 g·mol^{-1}, respectively.

The concentration of free cyclic carbonate groups in the **prepolymers** and **IFHEURs** was calculated based on their ^{1}H NMR spectra (e.g. **Fig. S6**) according to **Eq. S5**.

Eq. S5 $$Carbonate\ groups_{[mol\%]} = \frac{\frac{\int_{b,b'}}{n_{b,b'}}}{\frac{\int_{l}}{n_{l}}+\frac{\int_{b,b'}}{n_{b,b'}}} \cdot 100$$

The integrals ∫**b, b'** and ∫**l** correspond to the intensity of the **b, b'** and **l** signals (4.38-4.47 ppm and 1.73 ppm at **Fig. S6**, respectively) coming from $\mathbf{CH_{2cyclic}}$ and $\mathbf{CH_2}CH_2NHC(O)O$ groups, respectively. The numbers of protons in **b, b'** and **l** groups related to $\mathbf{n_{b,b'}}$ and $\mathbf{n_l}$ symbols and equalled **2.00** in both cases.

The contents of urethane groups in **prepolymers** and **IFHEURs** were calculated based on ^{1}H NMR spectra (e.g. **Fig. S18**) according to **Eq. S6**.

Eq. S6 $$Urethane\ groups_{[mol\%]} = \frac{\frac{\int_{d,d'}}{n_{d,d'}}}{\frac{\int_{d,d'}}{n_{d,d'}}+\frac{\int_{ethers}}{n_{ethers}}} \cdot 100$$

The integrals ∫**d, d'** and ∫**ethers** correspond with the intensity of the **d, d'** and **all ethers** signals (3.22 ppm and about 3.50-3.71 ppm at **Fig. S18**, respectively) coming from $\mathbf{CH_2}$ groups neighbouring to urethane groups and $\mathbf{CH_2}$ groups in ether groups, respectively. The numbers of protons in **d, d'** and **ethers** groups related to $\mathbf{n_{d,d'}}$ and $\mathbf{n_{ethers}}$ symbols and equalled **2.00** and **8.00**, respectively. It was assumed that the amounts of $\mathbf{CH_2}$ groups from ether groups in terminal groups and PRIAMINE structural units were negligible.

The content of unreacted amine groups in the obtained **IFHEURs** was calculated based on ^{1}H NMR spectra (e.g. **Fig. S18**) according to the **Eq. S7**.

Eq. S7 $$Amine\ groups_{[mol\%]} = \frac{\frac{\int_{y}}{n_{y}}}{\frac{\int_{y}}{n_{y}}+\frac{\int_{ethers}}{n_{ethers}}} \cdot 100$$

The integrals ∫**y** and ∫**ethers** correspond with the intensity of the **y** and **all ethers** signals (2.87 ppm and about 3.50-3.71 ppm at **Fig. S18**, respectively) coming from $\mathbf{CH_2}$ groups

neighbouring to amine groups and **CH_2** groups in ether groups, respectively. The numbers of protons in **y** and **all ethers** groups related to **n_y** and **n_{ethers}** symbols and equalled **2.00** and **8.00**, respectively.

5. Viscosity measurements in the online capillary viscometer of the extruder during REX synthesis of IFHEURs.

The viscosity of the reaction mixture during the REX synthesis was measured with the online capillary viscometer of the laboratory extruder Thermo Scientific HAAKE MiniLab II Micro Compounder. The backflow channel of the extruder is designed as a slit capillary with height of $\boldsymbol{h}$ = 1.5 mm, width of $\boldsymbol{w}$ = 10 mm, two pressure sensors at inlet ($\boldsymbol{P1}$, max. 200 bar) and outlet ($\boldsymbol{P2}$, max. 100 bar) of the capillary and the distance between the pressure sensors $\boldsymbol{P1}$ and $\boldsymbol{P2}$ of $\boldsymbol{L}$ = 64 mm (**Fig. S30**).

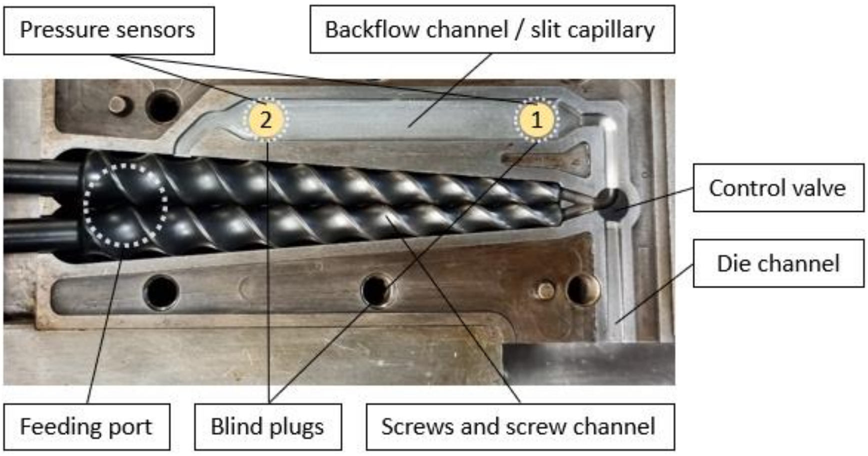

Fig. S30. Bottom section of the laboratory extruder with conical twin screws and a backflow channel. The dotted lines indicate where the feeding port and the bling plugs are located in the upper section of the extruder.

The channel is therefore functioning as an online capillary viscometer if the material is flowing in a circulation mode during the extrusion process. The measured pressure drop along the capillary is monitored online and recorded by the software PolySoft OS. The software calculates the shear stress ($\boldsymbol{\tau}$), apparent shear rate ($\dot{\boldsymbol{\gamma}}^*$) and apparent viscosity ($\boldsymbol{\eta_m^*}$) of the melt according to the **Eq. S8**, **Eq. S9** and **Eq. S10**, respectively:

Eq. S8 $$\tau_{[N\cdot m^{-2}]} = \left(\frac{h\cdot w}{2\cdot(h+w)\cdot L}\right)\cdot \Delta P$$

Eq. S9 $$\dot{\gamma}^*{}_{[s^{-1}]} = \left(\frac{6}{w\cdot h^2}\right)\cdot \dot{V}$$

Eq. S10 $$\eta_{m\ [Pa\cdot s]}^* = \frac{\tau}{\dot{\gamma}}$$

where the $\boldsymbol{h}$ is height the slit capillary in mm, the $\boldsymbol{w}$ is width of the slit capillary in mm, the $\boldsymbol{L}$ is the distance between the pressure sensors $\boldsymbol{P1}$ and $\boldsymbol{P2}$ in mm, the $\Delta\boldsymbol{P}$ is the recorded pressure drop in Pa, and the $\dot{\boldsymbol{V}}$ is the volume flow rate of the material in $mm^3 \cdot s$.

The $\dot{\boldsymbol{V}}$ through the capillary is correlated proportionally to the screw speed and the inlet pressure. Thus, the generated $\dot{\boldsymbol{\gamma}}^*$ and the resulting $\boldsymbol{\eta}_{\boldsymbol{m}}^*$ at a set screw speed is dependent on the flow properties of the melt. The processing conditions and the corresponding $\boldsymbol{\eta}_{\boldsymbol{m}}^*$ observed during exemplary **REX** synthesis of **PRE_1.1_PRI(1.0)** are shown in **Fig. S31**, while the final data at 120 min for all the obtained **IFHEUR** are shown in **Table S3**.

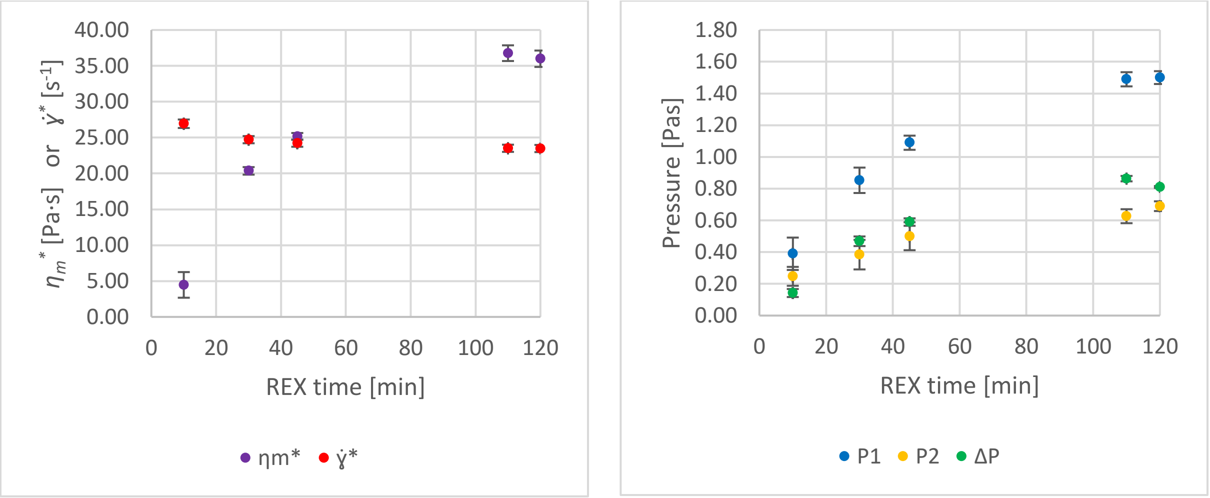

Fig. S31. The data obtained during the online monitoring of the **PRE_1.1_PRI(1.0)** during the **REX** synthesis. The average value and standard deviation are calculated based on 20 consecutive measurement points.

Table S3. The data obtained during the online monitoring of the **IFHEUR** at 120 min of **REX** synthesis. The average value and standard deviation are calculated based on 20 consecutive measurement points.

Sample	Melt temp. /°C	$\boldsymbol{P1}$ /bar	$\Delta\boldsymbol{P}$ /bar	$\dot{\boldsymbol{\gamma}}^*$ /s^{-1}	$\boldsymbol{\eta}_{\boldsymbol{m}}^*$ /Pa·s
**PRE_1.1_PRI(0.8)**	120	1.11±0.04	0.70±0.04	24.19±0.03	29.71±0.72
**PRE_1.1_PRI(0.9)**	120	1.50±0.03	0.79±0.02	23.46±0.03	33.73±1.44
**PRE_1.1_PRI(1.0)**	120	1.50±0.04	0.81±0.01	23.48±0.03	36.00±1.14
**PRE_1.1_PRI(1.0)_100C**	100	1.46±0.08	0.96±0.04	23.55±0.09	41.15±2.42
**PRE_1.2_PRI(1.0)**	120	0.60±0.03	0.41±0.01	25.76±0.09	15.55±0.61
**PRE_1.2_PRI(1.2)**	120	1.09±0.05	0.72±0.06	24.25±0.05	29.89±0.92
